# Diabetes alters immune response patterns to acute melioidosis in humans

**DOI:** 10.1002/eji.201848037

**Published:** 2019-05-08

**Authors:** Barbara Kronsteiner, Panjaporn Chaichana, Manutsanun Sumonwiriya, Kemajitra Jenjaroen, Fazle Rabbi Chowdhury, Suchintana Chumseng, Prapit Teparrukkul, Direk Limmathurotsakul, Nicholas P.J. Day, Paul Klenerman, Susanna J. Dunachie

**Affiliations:** ^1^ Centre for Tropical Medicine and Global Health University of Oxford Oxford UK; ^2^ Peter Medawar Building for Pathogen Research Nuffield Department of Medicine University of Oxford Oxford UK; ^3^ Mahidol‐Oxford Tropical Medicine Research Unit Mahidol University Bangkok Thailand; ^4^ Bangabandhu Sheikh Mujib Medical University (BSMMU) Dhaka Bangladesh; ^5^ Sunpasitthiprasong Hospital Ubon Ratchathani Thailand; ^6^ Department of Tropical Hygiene Faculty of Tropical Medicine Mahidol University Bangkok Thailand; ^7^ National Institute for Health Research Oxford Biomedical Research Centre University of Oxford Oxford UK

**Keywords:** CX3CR1, diabetes, melioidosis, NK cells, T cells

## Abstract

Diabetes mellitus (DM) is a serious global health problem currently affecting over 450 million people worldwide. Defining its interaction with major global infections is an international public health priority. Melioidosis is caused by *Burkholderia pseudomallei*, an exemplar pathogen for studying intracellular bacterial infection in the context of DM due to the 12‐fold increased risk in this group. We characterized immune correlates of survival in peripheral blood of acute melioidosis patients with and without DM and highlight different immune response patterns. We demonstrate the importance of circulating NK cells and show that CX3CR1 expression on lymphocytes is a novel correlate of survival from acute melioidosis. Furthermore, excessive serum levels of IL‐15 and IL‐18BP contribute to poor outcome independent of DM comorbidity. CD8^+^ T cells and granzyme B expression in NK cells are important for survival of non‐DM patients, whereas high antibody titers against *B. pseudomallei* and double‐negative T cells are linked to survival of DM patients. Recall responses support a role of γδ T‐cell‐derived IFN‐γ in the establishment of protective immunity in the DM group. Defining the hallmarks of protection in people with DM is crucial for the design of new therapies and vaccines targeting this rapidly expanding risk group.

## Introduction

Diabetes is a serious global health problem currently affecting over 450 million people worldwide 1 and causing an enormous economic burden 2. An increased risk of infection and poorer outcomes in type 2 diabetes (T2D) is seen for intracellular pathogens, with a threefold increased risk of developing tuberculosis (TB) 3, and increased risk of death or treatment failure in TB 4. Relationships between diabetes and a range of global pathogens have been reported, including Hepatitis B 5, Hepatitis C 6, and dengue 7. Defects in activation and function of innate immune cells, and a subsequent delay of IFN‐γ driven T cell responses have been implicated in the increased susceptibility to infection 8. The highest risk association by far (12‐fold) for an infectious disease and diabetes is seen for melioidosis 9, 10, a neglected tropical disease caused by the Gram‐negative facultative intracellular bacterium *Burkholderia pseudomallei* (*BP*). This soil‐dwelling pathogen is prevalent in Southeast Asia and Northern Australia, and is a major cause of mortality in these regions 11. It is increasingly recognized to be a significant but under‐reported cause of disease in other tropical regions worldwide, with a recent study integrating the environmental presence of *BP* and available case reports estimated 165 000 human melioidosis cases per year, causing 89 000 deaths 12. Aside from diabetes mellitus (DM), melioidosis is associated with other risk factors including chronic renal and lung disease, alcohol consumption, and increasing age 13, thus posing a challenge for vaccine design and immunomodulatory therapeutics targeting these at‐risk groups.

The bacterium can be transmitted by various routes (skin, inhalation, ingestion), and causes a wide spectrum of disease ranging from localized infection (pneumonia, abscesses) to systemic disease (liver, spleen, brain) and sepsis 14. Like many other intracellular pathogens, it is able to survive intracellularly in phagocytes including macrophages, neutrophils, and monocytes 15, 16. Recently, it has been demonstrated in vitro and in vivo that dendritic cells serve as vehicles for *BP* thus facilitating systemic dissemination 17. The importance of rapid innate immune responses and IFN‐γ production for the early control of melioidosis has been highlighted in several in vitro, animal, and clinical studies. Depletion of neutrophils resulted in acute infection in the otherwise chronic C57BL/6 mouse infection model 18 and depletion of macrophages led to significantly increased mortality in both acute (Balb/c) and chronic melioidosis mouse models 19, 20. Pre‐activation of macrophages with IFN‐γ in vitro has been shown to enhance their killing of *BP* due to an increase in iNOS 21. However, in vivo only IFN‐γ but not iNOS contributed to early protection in *BP*‐infected C57BL/6 mice 19. Blocking IFN‐γ in an experimental mouse model of melioidosis drastically lowered the LD50 dose and highly increased bacterial burden in liver and spleen, with extensive destruction of lymphoid architecture within just 2 days post infection 22.

Given the high in‐hospital case fatality rate, exceeding 40% in Northeast Thailand 10, the increasing rate of diabetes in melioidosis‐endemic areas 23, and the current lack of therapeutics and vaccines, melioidosis poses a major global health threat. Despite advancements in the understanding of immune responses to melioidosis, there is still a lack of knowledge on cellular immune responses in acute human melioidosis with diabetes comorbidity. The unparalleled susceptibility relationship between diabetes and melioidosis presents a unique model to study the influence of diabetes in intracellular bacterial infection. Thus, the aim of this study was to characterize the composition of PBMCs and serum cytokines of acute melioidosis patients, who survived and died, in order to identify commonalities and differences in the correlates of survival between patients with DM and without (non‐DM). Furthermore, the influence of diabetes comorbidity on the induction of protective immune responses was studied in recovered melioidosis patients with and without DM. To our knowledge this is the first comprehensive report on the immune cell phenotype during acute melioidosis in humans.

## Results

### Patient characteristics

135 acutely ill patients enrolled in a longitudinal study of melioidosis in Northeast Thailand were studied for immunophenotyping and cytokine profiling to define novel immune correlates of survival and differences based on the presence (DM) or absence (non‐DM) of diabetes. Characteristics of the patient cohort and endemic control subjects are displayed in Table 1. DM survivors were characterized by significantly higher indirect hemagglutination assay (IHA) antibody titers compared to DM fatal cases. Although there was no difference in HbA1c levels based on outcome, the acute melioidosis group with DM showed significantly worse glycemic control compared to the endemic control group, as reported previously 24. Melioidosis patients without DM were comprised of a heterogeneous population of individuals with one or more risk factors other than diabetes 25. Renal failure was highly associated with death in this patient group. Full blood counts (Table 1) taken on admission to the hospital showed lymphopenia in died compared to survived patients regardless of diabetes comorbidity, which is typically seen in early acute sepsis 26 due to the recruitment of circulating lymphocytes to the site of infection and apoptotic depletion. Interestingly, neutrophil counts were significantly increased in died compared to survived non‐DM patients, and monocyte counts were significantly lower in died compared to survived DM patients. In order to define phenotypic changes upon acute melioidosis infection, PBMC from melioidosis patients with and without DM, who survived or died, as well as endemic controls were studied for the presence of common immune cell populations. Overall, viability of PBMC isolated from non‐DM patients, who died, was significantly decreased with an increased frequency of dead and late apoptotic cells. Evaluation of cell subsets within early apoptotic cells revealed an increased frequency of classical monocytes suggesting that these cells preferentially underwent apoptosis in fatal non‐DM cases of acute melioidosis (data not shown).

**Table 1:** Patient characteristic and differential blood count of melioidosis and endemic control cohort for immunophenotyping of PBMC

	Melioidosis cohort						Endemic control cohort			
	No Diabetes (non‐DM)			Diabetes (DM)			non‐DM		DM	
Characteristic	Survived (*n* = 19)	Died (*n* = 15)	*p*‐Value^c^	Survived (*n* = 19)	Died (*n* = 20)	*p*‐Value^c^	(*n* = 14)	*p*‐Value^d^	(*n* = 15)	*p*‐Value^d^
Age (years), mean (range)	54 (41–68)	61 (33–79)	0.05	53 (41–64)	55 (37–69)	0.52	52 (41–73)	0.13	58 (51–65)	0.07
Sex M/F (%M)	15/4 (78.9)	11/4 (73.3)	0.96	10/9 (52.6)	11/9 (55.0)	0.89	9/5 (64.3)	0.40	12/3 (80.0)	0.08
HbA1c (%), mean (range)	5.5 (4.3–6.9)	5.8 (4.9–6.6)	0.25	10.7 (6.7–12.8)	10.0 (5.5–13.2)	0.25	NA^b^	NA	7.1 (5.2–9.7)	<0.001
IHA titre, median (IQR)	1:80 (1–160)	1:80 (1–640)	0.70	1:640 (160–1280)	1:80 (10–320)	0.008	<1:40		<1:40	
No. (%) with bacteremia	5 (26.3)	11 (73.3)	0.01	6 (31.6)	17 (85.0)	0.001				
No. (%) with renal disease	2 (10.5)	8 (53.3)	0.005	4 (21.1)	4 (20.0)	0.94				
No. (%) with ≥1 other risk factor^a^	7 (36.8)	5 (33.3)	0.86	4 (21.1)	1 (5.0)	0.13				
Neutrophils (per microliter blood), median (IQR)	6900 (4640–10329)	12357 (10960–19701)	0.009	9620 (7260–13348)	11932 (9156–14210)	0.20				
Monocytes (cells per microliter blood), median (IQR)	8234 (505.4–1238)	418.8 (271.6–1372)	0.23	795.6 (578.4–1166)	509.2 (290.7–648.9)	0.006				
Lymphocytes (cells per microliter blood), median (IQR)	1743 (1146–2628)	814.8 (349.8–1872)	0.02	1915 (1326–2744)	973 (565.1–1578)	0.002				

a Excluding diabetes and renal disease, including liver disease, lung disease, heart disease, cancer, and alcoholism.

b Unfortunately, this information is not available due to limited resources.

c Comparing survived and died.

d Comparing melioidosis and endemic cohort.

### T and NK cells are important for survival from acute melioidosis irrespective of diabetes status

**Figure 1:**
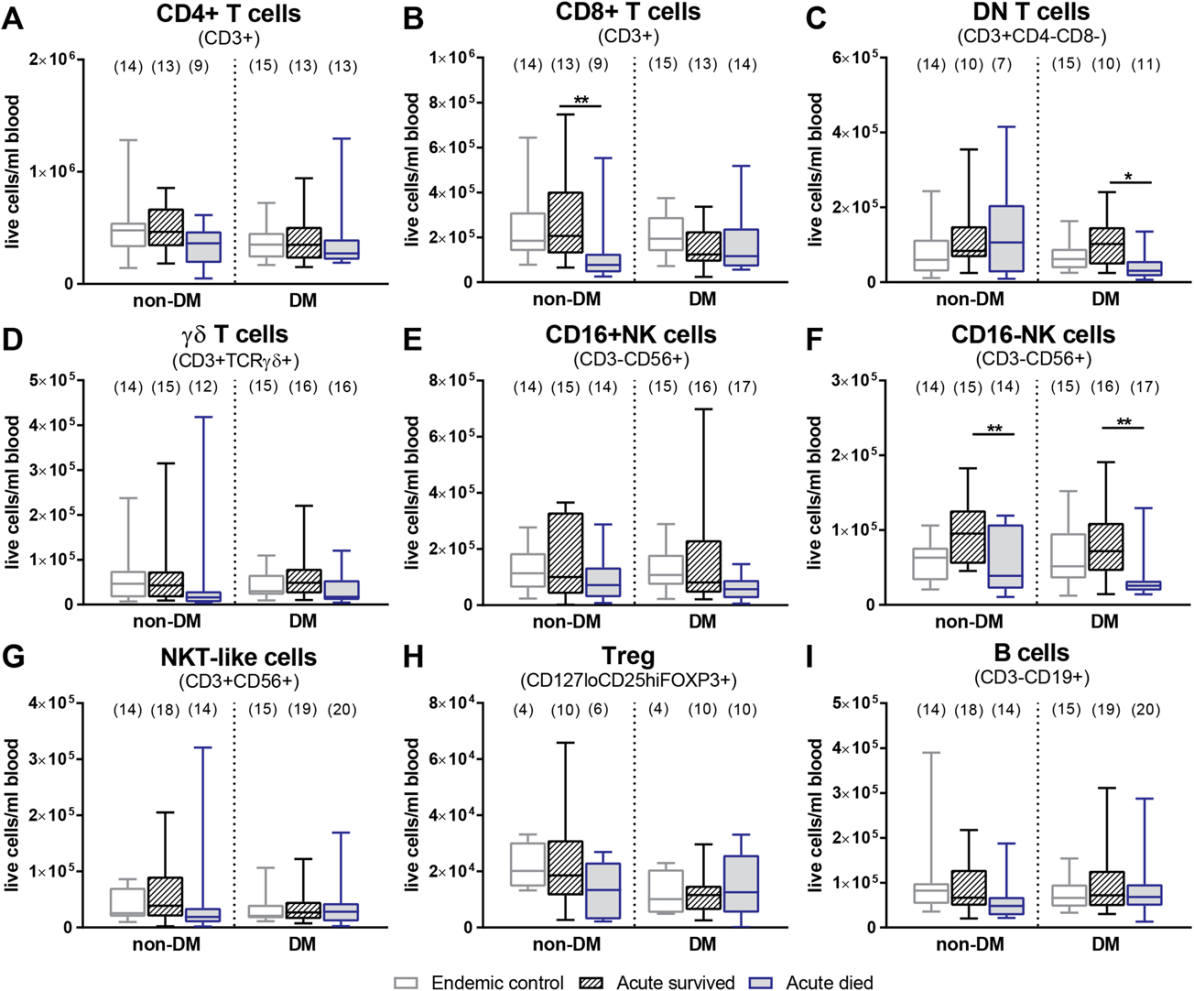
Circulating immune cell subsets during acute melioidosis. The absolute frequency (cells per milliliter blood) of (A) CD4^+^ T cells, (B) CD8^+^ T cells, (C) CD4^−^CD8^−^ double negative (DN) T cells, (D) γδ T cells, (E) CD16^+^ NK cells, (F) CD16^−^ NK cells, (G) NKT‐like cells, (H) Tregs, and (I) B cells was determined by multicolor flow cytometry in PBMC from acute melioidosis patients with (DM) and without diabetes (non‐DM) who survived (black, striped) and died (blue, filled) as well as endemic controls (grey). Six experiments with three to four biological replicates per group were performed. Total number of biological replicates per group is given in brackets on top of blots. Data are presented in box and whiskers blots. Mann–Whitney *U* test was performed on survived versus died groups and significant differences are depicted as *p ≤ 0.05 and **p ≤ 0.01. The gating strategy used is described and shown in detail in the MIFlowCyt file provided as Supporting Information.

IFN‐γ has been previously implicated in survival from acute melioidosis, with T cells and NK cells being the main producers 24, 27, 28, 29, 30. Therefore, we evaluated differences in absolute (number of live cells per milliliter of blood; Fig. 1; Supporting Information Table 1) and relative frequencies (percentage of live cells, Supporting Information Fig. 1) of lymphocyte populations in non‐DM and DM patients based on outcome (28‐day mortality). Endemic control cohorts with and without DM were included to provide reference values in the absence of infection. Acute melioidosis patients who died compared to survivors had significantly less circulating CD16^−^ NK cells irrespective of DM comorbidity (Fig. 1F, Supporting Information Fig. 1F). Interestingly, this correlated positively with bacterial antibody titers (*r* ^2^ = 0.5, *p* = 0.005, Spearman's rank correlation) in the DM group alone. A trend for lower CD4^+^ T cell numbers (Fig. 1A, Supporting Information Table 1) and a significant reduction of the CD8^+^ T cell subset (Fig. 1B, Supporting Information Table 1) was observed in died compared to survived non‐DM patients overall resulting in a significant reduction of total T cells (Supporting Information Table 1). In contrast, fatal cases in the DM group had significantly lower numbers of CD4^−^CD8^−^ double negative (DN) T cells compared to survivors (Fig. 1C, Supporting Information Table 1). Within the DM cohort we found a mild correlation of HbA1c (%) to the number of total circulating lymphocytes and specifically DN, CD8^+^ and γδ T cells (all *r* ^2^ = 0.4, *p* ≤ 0.05, Spearman's rank correlation). No differences in the frequency of γδ T cells, CD16^+^ NK cells, NKT‐like cells, Tregs, and B cells between died and survived patients were found (Fig. 1D, E, G–I, Supporting Information Fig. 1B, C, E–G) in either group.

### Cytotoxic effector properties of NK cells are linked to survival in patients without diabetes

**Figure 2:**
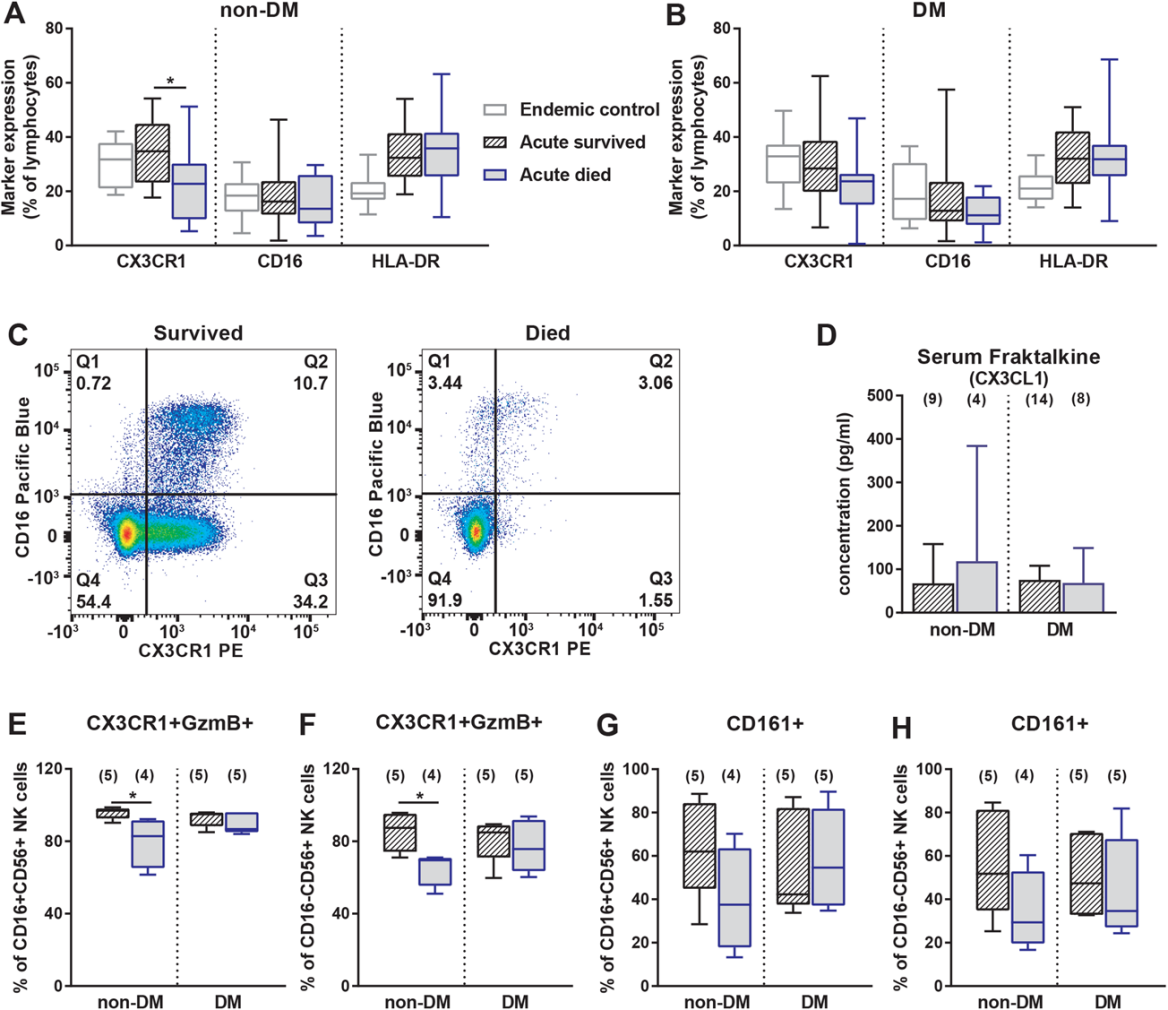
CX3CR1 and GzmB is reduced in fatal cases of acute melioidosis without diabetes. Expression of CX3CR1, CD16, and HLA‐DR was measured by flow cytometry on total lymphocytes of survived (black‐striped, *n* = 18–19) and died (blue‐filled, *n* = 14–20) acute melioidosis patients (A) without (non‐DM) and (B) with diabetes (DM). Six experiments with three to four biological replicates per group were performed. (C) Representative flow cytometry dot blots show CX3CR1 and CD16 expression on lymphocytes of one survived and one died melioidosis patients without DM. Numbers in quadrants represent percentage of lymphocytes. (D) Serum fraktalkine (CX3CL1) levels were determined in acute melioidosis patients with and without DM, who survived (black‐striped) and died (blue‐filled). One experiment was performed with *n* = 4–14/group. CX3CR1 and Granzyme B (GzmB) co‐expression as well as CD161 expression were measured on (E, G) CD16^+^ and (F, H) CD16^−^ NK cells, respectively. One experiment was performed with four to five biological replicates per group. Phenotypic data is presented in box and whiskers blots and cytokine concentration is presented as median with 95% confidence interval. Mann–Whitney *U* test was performed comparing survived and died groups and significant differences are depicted as *p ≤ 0.05. The gating strategy used is described and shown in detail in the MIFlowCyt file provided as Supporting Information.

Due to the nature of *BP* being an intracellular pathogen, cytotoxic lymphocytes are likely to be important for the control of infection. The chemokine receptor CX3CR1 regulates leukocyte trafficking at the vascular endothelium 31 and has been shown to specifically promote migration of cytotoxic effector lymphocytes to sites of inflammation 32. In addition, binding of CX3CR1 to its ligand fraktalkine (CX3CL1) expressed by mature DC is important for activation of resting NK cells 33. Given the decreased frequency of T and NK cells in acute melioidosis, we were interested in the expression of this chemokine receptor on lymphocytes and its role in survival. The relative frequency of lymphocytes (CD3^+^, CD19^+^, CD20^+^, CD56^+^) expressing CX3CR1 (Fig. 2A and B) as well as absolute frequency of CX3CR1^+^ lymphocytes (Supporting Information Table 1) were significantly suppressed in died compared to survived individuals without DM only (Fig. 2C), and no difference in the expression of CD16 and HLA‐DR was observed (Fig. 2A and B). Fraktalkine was not differentially expressed in serum of a subset of acute melioidosis patients with and without DM (Fig. 2D).

CX3CR1 is expressed on 10–30% of CD3^+^ T cells, ∼70% of γδ T cells, and the majority of CD3^−^CD56^+^ NK cells 32. No difference in CX3CR1 expression on CD4^+^, CD8^+^, DN, and γδ T cells (Supporting Information Fig. 2A–D) was found in survived compared to died non‐DM and DM patients. In contrast, we observed a significant reduction of NK cells expressing CX3CR1 and Granzyme B (GzmB, Fig. 2E and F) and a trend for decreased expression of CD161 (Fig. 2G and H) in died compared to survived non‐DM patients. Furthermore, died non‐DM patients also expressed lower levels of CX3CR1 and GzmB on CD16^+^ (median MFI CX3CR1: 490 vs. 754, *p* = 0.11, median MFI GzmB: 2454 vs. 3967, *p* = 0.02) and CD16^−^ NK cells (median MFI CX3CR1: 393 vs. 618, *p* = 0.06, median MFI GzmB: 2444 vs. 3604, *p* = 0.02) compared to survivors. Overall this suggests a functional defect of NK cells in the non‐DM group.

### Elevated levels of IL‐15 and biologically inactive IL‐18 are linked to poor outcome

**Figure 3:**
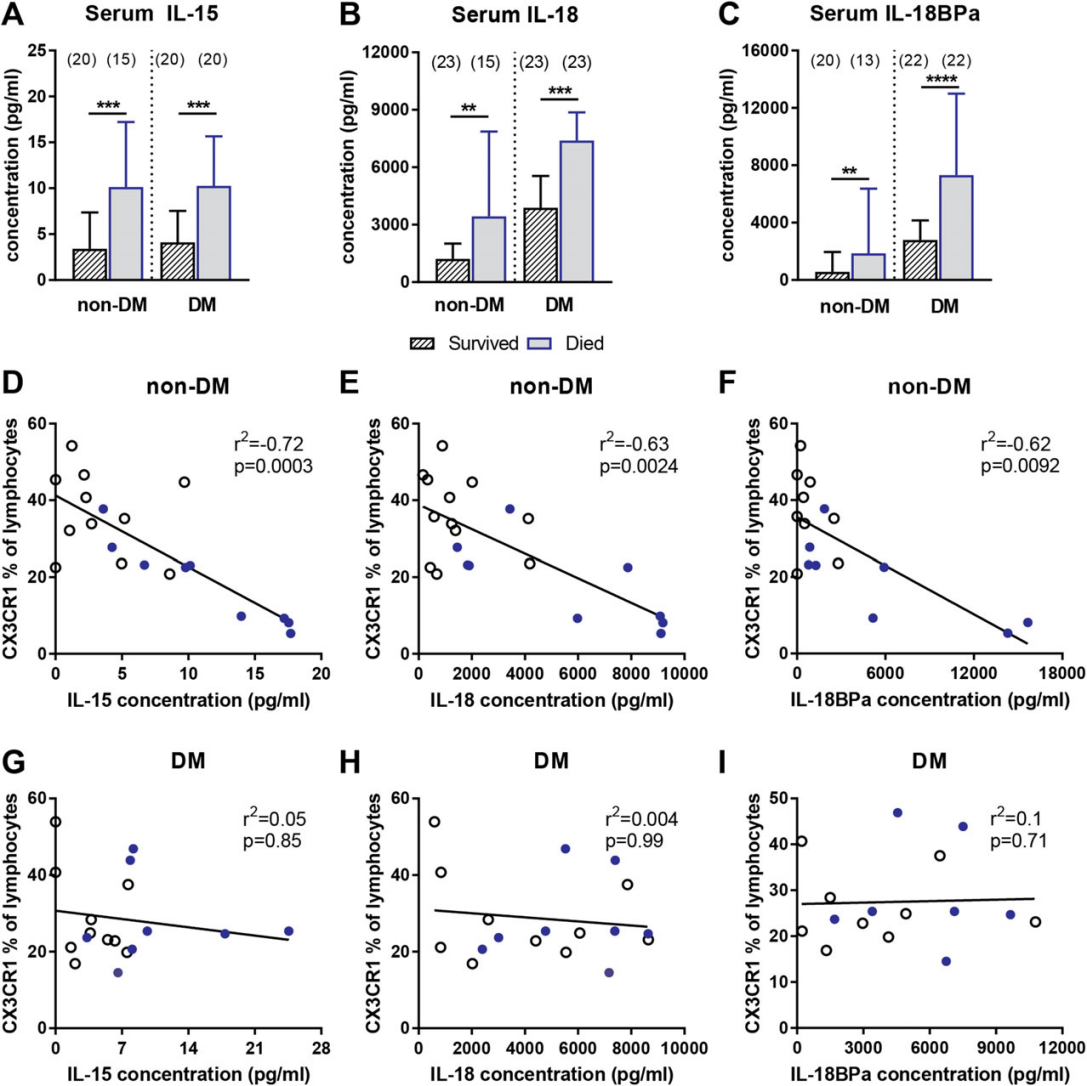
Serum cytokines relevant for NK cell development and effector function. Levels of (A) IL‐15, (B) IL‐18, and (C) IL‐18/IL‐18BPa complex were determined by ELISA using serum of acute melioidosis patients with (DM) and without (non‐DM) diabetes, who survived (black‐striped) and died (blue‐filled). One experiment with 13–23 biological replicates per group. Exact number per group is given in brackets on top of blots. Data is presented as median with 95% confidence interval. Mann–Whitney *U* test was performed to compare survived and died groups, and significant differences are depicted as **p ≤ 0.01, ***p ≤ 0.001, ****p ≤ 0.0001. Spearman correlation analysis was performed interrogating the relationship of cytokine concentration with CX3CR1 expression in (D–F) non‐DM and (G–I) DM patients. Open, black dots: survived; filled, blue dots: died.

To assess whether changes in the expression of cytotoxicity markers on NK cells and the overall reduction of this cell subset were due to alterations of key cytokines involved in their function and proliferation, we next measured serum levels of IL‐15, IL‐18, and IL‐18/IL‐18BPa complex. IL‐18 has previously been correlated to mortality in acute melioidosis 34. It is produced by APCs and together with IL‐12 and IL‐15 promotes IFN‐γ production by T and NK cells 35, 36. IL‐18/IL‐18BPa measures IL‐18 bound to IL‐18 binding protein, thus making it biologically unavailable 37. All of these cytokines were significantly increased in died acute melioidosis patients irrespective of diabetes comorbidity (Fig. 3A–C). However, correlation analysis between CX3CR1 expression on lymphocytes and cytokine levels revealed a strong inverse correlation for non‐DM patients only (Fig. 3D–F, IL‐15: *r* ^2^ = –0.7, *p* = 0.0003; IL‐18: *r* ^2^ = –0.6, *p* = 0.002; IL‐18/IL‐18BPa: *r* ^2^ = –0.6, *p* = 0.009). Furthermore, a comparison of melioidosis patients based on diabetes status alone revealed significantly higher levels of IL‐18 (*p* < 0.0001) and IL‐18BPa (*p* < 0.0001) in the circulation of DM compared to non‐DM patients with acute melioidosis. A multivariable regression model showed that CX3CR1 expression on lymphocytes is an independent predictor of death from acute melioidosis, when controlling for age, sex, diabetes status, and preexisting renal disease. Patients expressing CX3CR1 on less or equal than 21.8% (25th percentile of survivors) of their lymphocytes had a threefold increased risk of death (*p* = 0.048; Table 2).

**Table 2:** Uni‐ and multivariable analysis for prediction of 28‐day mortality in patients with acute melioidosis

	Univariable	Multivariable	
Variables	*p*‐Value	Adjusted Odds Ratio (95% CI)	*p*‐Value
Age	0.06	1.06 (0.99–1.13)	0.09
Sex	0.99	1.06 (0.34–3.26)	0.92
Diabetes (HbA1c ≤ 7%)	0.53	1.91 (0.65–5.63)	0.24
Preexisting renal disease	0.06	2.56 (0.73–8.90)	0.14
CX3CR1 percentage of lymphocytes (≤21%)^a^	0.13	3.18 (1.01–9.99)	0.05
Intermediate monocytes (≥19 821 cells/mL blood)^b^	0.44	1.22 (0.40–3.70)	0.73

a Cutoff was set below the 25% percentile of survivors

b Cutoff was set above the 75% percentile of survivors

So far, we have demonstrated that acute melioidosis patients irrespective of DM status rely on NK cells for survival with CX3CR1 and GzmB being important in non‐DM patients only. We further highlight differences in the contribution of T cell subsets to survival in non‐DM and DM patients with CD8^+^ and DN T cells being important, respectively.

### The role of antigen presenting cells in acute melioidosis

**Figure 4:**
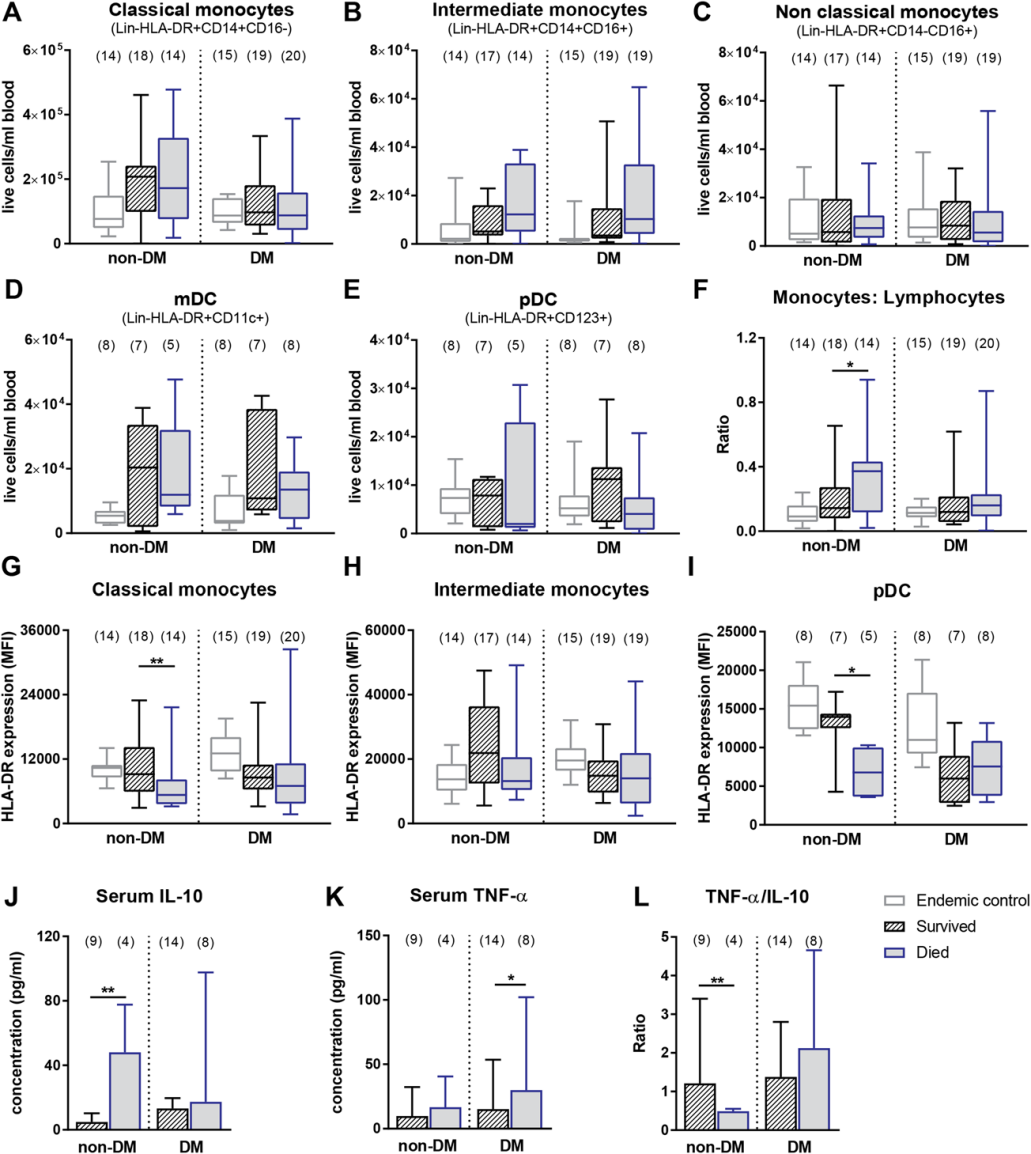
The role of APCs in acute melioidosis. The absolute frequency (cells per milliliter blood) of (A) classical, (B) intermediate, (C) and nonclassical monocytes, (D) plasmacytoid (p) and (E) myeloid (m) dendritic cells (DC) as well as (F) the ratio of monocytes to lymphocytes was determined by multicolor flow cytometry in PBMC from acute melioidosis patients with (DM) and without (non‐DM) diabetes, who survived (black, striped) and died (blue, filled) as well as endemic controls (grey). Surface expression of HLA‐DR was assessed as median fluorescence intensity (MFI) on (G) classical monocytes, (H) intermediate monocytes, and (H) pDC in the same patient groups. The gating strategy used is described and shown in detail in the MIFlowCyt file provided as Supporting Information. Six experiments with three to four biological replicates per group in case of monocytes and two experiments with three biological replicates per group in case of DC. Data are presented in box and whiskers plots. Serum levels of (J) IL‐10 and (K) TNF‐α and (L) the ratio of TNF‐α to IL‐10 were determined in DM and non‐DM acute melioidosis patients, who died and survived. One experiment was performed with 4–14 biological replicates per group. Data are presented as median with 95% confidence interval. The exact number of biological replicates per group is given in brackets on top of blots. Mann–Whitney *U* test was performed comparing survived versus died groups and significant differences are depicted as *p ≤ 0.05 and **p ≤ 0.01.

Since APCs are crucial for the initiation of effector and memory T cell responses, we next assessed the importance of this innate cell compartment in survival from acute melioidosis and show a trend for increased intermediate monocytes (Fig. 4B, Supporting Information Fig. 1K) in died compared to survived non‐DM and DM patients, respectively. Numbers of myeloid dendritic cells (mDC), plasmacytoid DC (pDC), classical monocytes, and nonclassical monocytes remained unchanged (Fig. 4A, C–E, Supporting Information Fig. 1H–J and L).

An imbalance in the ratio of monocytes to lymphocytes (either too low or too high) is associated with active tuberculosis 38, and was identified as a correlate of risk for tuberculosis 39. In acute melioidosis, the combination of decreased lymphocytes (Supporting Information Table 1) together with increased monocytes (Supporting Information Table 1) resulted in a dramatically increased monocyte to lymphocyte ratio (Fig. 4F) in died compared to survived non‐DM patients only. Downregulation of the antigen presentation molecule HLA‐DR on monocytes has been previously associated with poor outcome in patients with systemic inflammatory response syndrome 40. We were able to link poor outcome in non‐DM melioidosis patients to a reduction in HLA‐DR expression on classical monocytes (Fig. 4G, Supporting Information Table 1). No changes were observed in the intermediate monocytes (Fig. 4H). Similar results were obtained when looking at pDC, which had significantly suppressed HLA‐DR expression (Fig. 4I, Supporting Information Table 1) and concomitantly increased CD86 expression (data not shown) in died compared to survived non‐DM patients. Such an inverse relationship of reduced MHC‐II and increased CD86 expression has been previously observed in murine pDC infected with *BP* 41. Since HLA‐DR is known to be downregulated in the presence of IL‐10 42, we sought to determine IL‐10 levels in serum of acute melioidosis patients. Corresponding to lower HLA‐DR expression, our cytokine results show significantly increased levels of IL‐10 in serum of died compared to survived non‐DM patients, which was not the case in the DM group (Fig. 4J). The ratio of TNF‐α/IL‐10 (Fig. 4L) was also significantly lower in died compared to survived non‐DM patients. In the case of DM patients, serum TNF‐α levels (Fig. 4K) were significantly higher in died compared to survived patients. However, the TNF‐α /IL‐10 ratio remained unchanged in the DM group. In general, IL‐10 levels were significantly higher in DM compared to non‐DM survivors (*p* = 0.02), which was corroborated in plasma using a larger patient cohort 43. In line with this, HLA‐DR expression on pDC was significantly lower in DM compared to non‐DM survivors (*p* = 0.01) and in contrast to non‐DM patients no further decrease of HLA‐DR expression was observed in DM patients, who died (Supporting Information Table 1). Antigen presentation via HLA‐DR is important for efficient activation and proliferation of T cells. Thus, the downregulation of HLA‐DR expression on APCs might result in less efficient T cell priming and subsequently impaired function. Therefore, we assessed the expression of exhaustion markers and proliferation of T cells. We did not find differences in PD‐1 and CTLA‐4 expression on any T cell subsets (data not shown). Regardless of outcome, T cells had equal proliferative capacity upon acute infection as demonstrated by positivity for the intracellular marker Ki‐67 (Supporting Information Fig. 3). The highest proportion of proliferating cells was found within the DN T cell population followed by CD8^+^ and CD4^+^ T cells. Of note, the proportion of proliferating CD8^+^ T cells was significantly lower in DM compared to non‐DM patients (median = 7% vs. 16.3%, *p* = 0.02) regardless of outcome.

### Rapid recall IFN‐γ responses by γδ T cells dominate in recovered melioidosis patients with diabetes

**Figure 5:**
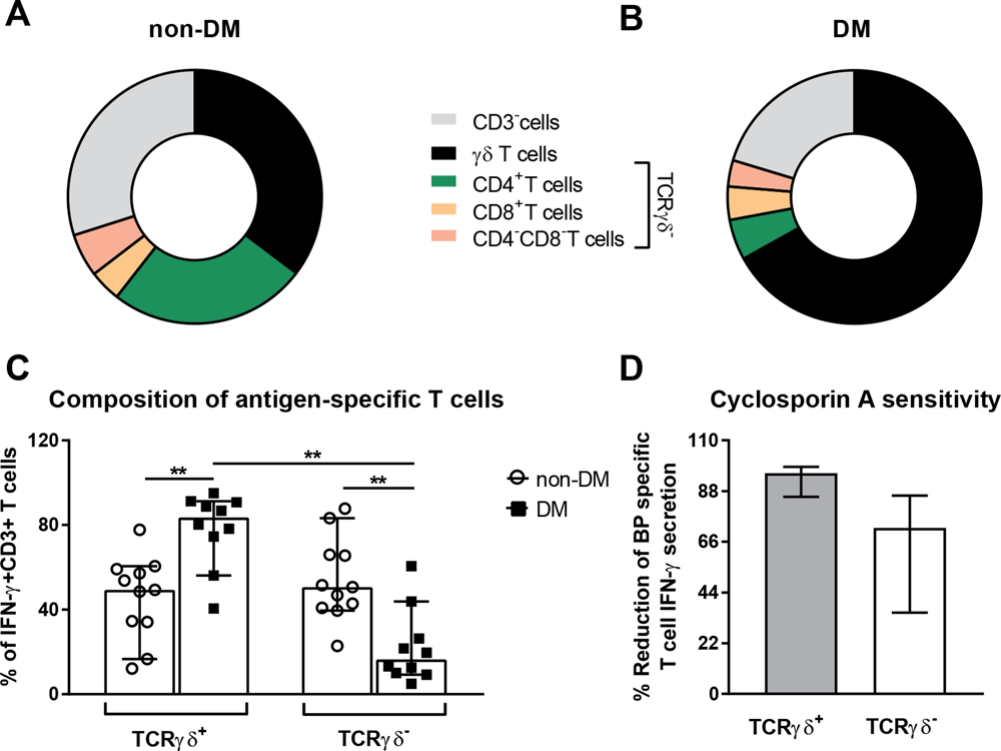
Contribution of γδ T cells to the early recall IFN‐γ response to *BP* antigens. The contribution of classical (TCRγδ^−^) T cell subsets, nonclassical γδ T cells, and CD3^−^ cells to total IFN‐γ secretion was assessed by multicolor flow cytometry upon performing an intracellular cytokine secretion assay on PBMCs from (A) non‐DM and (B) DM melioidosis patients within 1 year post enrolment. The pie charts show a mean of *n* = 10–11 biological replicates per group. (C) The contribution of TCRγδ^+^ and TCRγδ^–^ cells to T cell derived IFN‐γ secretion was evaluated by flow cytometry in the same patients as described above. Mann–Whitney U test was used for unpaired data and Wilcoxon test for paired data with significant differences depicted as **p ≤ 0.01. Five experiments with two to three biological replicates per group, total *n* = 10–11/group. (D) The effect of cyclosporine A (CsA) on T cell derived IFN‐γ secretion upon antigenic stimulation was measured by flow cytometry in a subset of recovered melioidosis patients with and without DM (one experiment, *n* = 5/group). Data is expressed as percentage (%) reduction of IFN‐γ secretion upon CsA treatment compared to DMSO‐treated cells. Wilcoxon test did not show significant differences. (C and D) Data are presented as median with 95% confidence interval. The gating strategy used is described and shown in detail in the MIFlowCyt file provided as Supporting Information.

In addition to differences in the immune responses during acute melioidosis, we were interested in the nature of recall IFN‐γ responses to *BP*. We have previously shown that IFN‐γ ELISpot responses were durable but did not significantly differ between non‐DM and DM patients 1 year post infection 24. Here, we performed intracellular cytokine analysis on PBMC stimulated with *BP* heat‐inactivated antigens and analyzed the contribution of T cell subsets and CD3^−^ cells to the IFN‐γ response 6 h post stimulation. Our data indicate a significant predominance of γδ T cell‐derived IFN‐γ in the recovered DM group and a mixed CD4^+^ and γδ T cell response in the non‐DM group within 1 year post infection (Fig. 5A–C). To demonstrate whether the observed IFN‐γ production by nonclassical γδ T cells in response to *BP* was TCR‐mediated or a cytokine driven bystander effect, PBMCs were treated with CsA or DMSO in the presence of *BP* antigens. CsA highly abrogated T cell specific IFN‐γ secretion in both classical and nonclassical T cells with a median reduction of 72% (IQR 47–83%) and 96% (IQR 89–98%), respectively (Fig. 5D).

## Discussion

Melioidosis is a neglected tropical disease with high prevalence in South East Asia and Northern Australia but predicted to be vastly underreported in other tropical regions of the world 12. While immunocompetent people typically defend themselves against the bacterium and do not get ill 44, 45, people with DM are unusually susceptible (12‐fold increased risk in diabetes 9, 10). With more than 50% of melioidosis cases having diabetes 46 and the rising incidence of diabetes worldwide 23, this bacterial disease poses a severe economic and public health threat in *BP* endemic regions.

**Figure 6:**
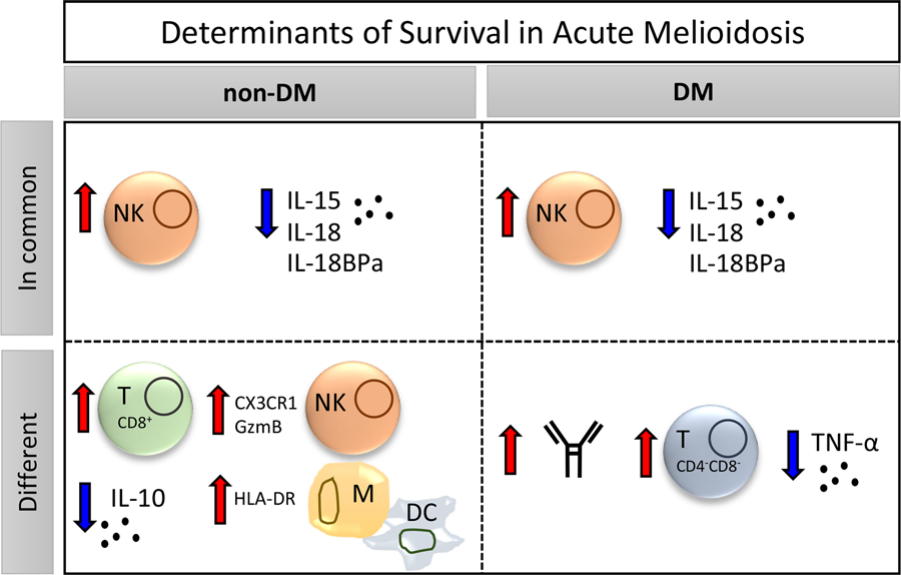
Determinants of survival in acute melioidosis. A schematic representation of in‐common and differing immune responses important for survival in acute melioidosis patients with (DM) and without (non‐DM) diabetes. Arrows next to cell types and soluble/surface molecules indicate changes that are increased (red) and decreased (blue) in survivors compared to patients who died. DC, dendritic cell; M, monocyte; NK, natural killer cell; blue arrow, decreased; red arrow, increased.

In this study, we demonstrate that acute melioidosis patients rely on NK cells for survival and excessive levels of pro‐inflammatory cytokines IL‐15 and biologically inactive IL‐18 contribute to poor outcome independent of diabetes comorbidity (Fig. 6). Although the levels of total IL‐18 were elevated in the serum of died patients, the concentration of IL‐18 bound in a complex with IL‐18BPa was also highly increased suggesting that IL‐18 is biologically inactive. This is in line with previous findings by Wiersinga et al. showing that increased levels of IL‐18BPa correlate with mortality in acute melioidosis 34. We further identify different immune response patterns in patients with and without diabetes (Fig. 6). Poor outcome in non‐DM acute melioidosis patients was not only associated with reduced numbers of NK cells but also with a downregulation of the chemokine receptor CX3CR1. CX3CR1 can be used to discriminate between intermediary CX3CR1^neg^CD56^dim^ and fully mature CX3CR1^hi^CD56^dim^ NK cells and may link NK cell maturation with their ability to migrate to different organs 47. IL‐15, which has previously been reported to be a negative regulator of CX3CR1 expression and function on NK cells 48, was significantly increased in died compared to survived melioidosis patients. Indeed, our data suggest functional impairment of NK cells in fatal cases characterized by suppression of GzmB and CD161, a molecule that characterizes a functionally distinct subset of pro‐inflammatory NK cells responsive to innate cytokines (IL‐12 and IL18) 49. Of note, we found a strong inverse correlation among IL‐15, IL‐18, and IL18BPa serum levels with CX3CR1 expression on lymphocytes in non‐DM patients only. CX3CR1 expression on lymphocytes was identified as a novel correlate of survival from acute melioidosis when considering all patients and controlling for diabetes. To our knowledge, this is the first report of CX3CR1 as a correlate of survival in infectious diseases. CX3CR1 is expressed on NK cells as well as DN, CD8^+^, and γδ T cells. It is likely that these cells and the expression of CX3CR1 will be important for survival from other intracellular bacterial infections and future studies should seek to examine this.

In addition, fatal cases in the non‐DM group showed signs of hypo‐inflammatory response syndrome typically observed in sepsis 50. Highly increased serum IL‐10 levels, a decreased TNF‐α/IL‐10 ratio, and the associated downregulation of HLA‐DR on monocytes and DC were linked to poor outcome only in patients without DM comorbidity. Furthermore, we show a significant decrease of CD8^+^ T cell numbers in non‐DM fatal cases, a T cell subset that has been implicated in the production of IFN‐γ in response to *BP* 24, 30.

In contrast, individuals with DM comorbidity who died had significantly reduced numbers of circulating NK cells without changes in cytotoxicity marker expression compared to survived patients. The significant decrease of DN T cells observed in DM fatal cases indicates a role for these cells in survival. DN T cells have been previously identified as important mediators for the control of intracellular bacterial infection in mouse models of *Francisella tularensis* and *M. tuberculosis* infection 51, which warrants further investigation in the context of melioidosis and DM comorbidity. In addition to these cellular changes, we measured significantly higher bacterial antibody titers in DM survivors of acute melioidosis as previously reported in Australia 52 and Thailand 53, and this correlated to the number of circulating NK cells. In some endemic regions, up to 80% of children produce antibodies to *BP* by the age of 4 years 54. Although there is currently no evidence that this is protective 24, both humoral and cell‐mediated immune responses were found to be necessary for optimal protection in a mouse vaccination model of melioidosis 55. The production of antibodies to *BP* in endemic regions could stem from environmental exposure to nonpathogenic *Burkholderia* species, such as *B. thailandensis* 56, and current work in our laboratory is evaluating such cross‐reactivity. Nevertheless, it is unclear why survivors with DM produce more antibodies to *BP*. Based on our data we can hypothesize that antibody‐dependent cellular cytotoxicity mediated by NK cells serves as an early response mechanism to eliminate infected host cells, reduce bacterial burden, and thus protect from death in people with DM during acute melioidosis.

We have previously demonstrated the importance of T cell IFN‐γ responses for survival of acute melioidosis in humans, with overall IFN‐γ responses being lower in DM compared to non‐DM patients 24. In this study, we identified possible contributors of the impaired IFN‐γ responses observed in acutely infected individuals with DM. Although circulating TNF‐α levels were significantly increased in DM fatal cases, the ratio of TNF‐α/IL‐10 was not elevated and this was due to a concomitant increase in IL‐10 levels. A comparison of DM and non‐DM acute melioidosis patients independently of outcome revealed higher serum levels of anti‐ and pro‐inflammatory cytokines (IL‐10, total IL‐18, and IL‐18BPa), a reduced frequency of proliferating CD8^+^ T cells and lower HLA‐DR expression on pDC in individuals with DM. Impaired early inflammatory cytokine production (IL‐1β, IL‐12, IFN‐γ, IL‐6, and TNF‐α) leading to uncontrolled bacterial growth has been previously demonstrated in a polygenic diet‐induced mouse model of T2D infected with *BP* 57. Furthermore, the stimulation of whole blood from individuals suffering from T2D with *BP* also revealed lower IFN‐γ and higher IL‐10 responses, with CD3^−^CD14^+^ monocytes being the main producers of IL‐10 58. As a group, the acute DM cohort had very poor glycemic control compared to DM outpatients. Although there was no association with mortality, highly elevated HbA1c levels might further contribute to susceptibility to infection due to sustained cellular metabolic changes. Indeed, PBMCs from diabetes patients with poor glycemic control that have been infected with *BP* or *M. tuberculosis* show impaired IL‐12p70 and IFN‐γ secretion, which is associated with poor bacterial killing and correlated with a deficiency in intracellular glutathione 59.

While the rapid IL‐12 and IL‐18 mediated production of IFN‐γ by CD8^+^ T cells and NK cells was found essential for the control of acute infection 30, 60, CD4^+^ T cells but not CD8^+^ T cells are essential for protection in immunized Balb/c mice 60. Our studies on protective memory T cell responses inducible up to 1 year post infection revealed substantial qualitative differences between patient groups. Early recall T cell responses revealed a predominance of γδ T cell‐derived IFN‐γ in diabetes patients, whereas mixed CD4^+^ and γδ T cell‐derived IFN‐γ responses prevailed in non‐DM. Phosphoantigen‐specific expansion of human γ9δ2 and marmoset γ9 T cells has previously been induced in vitro using heat killed *BP* and synthetic phosphoantigen‐stimulated PBMCs were able to reduce bacterial load in *BP*‐infected monocytes, providing evidence for a role of γδ T cells in melioidosis 61. However, it is not yet clear, why recovered melioidosis patients with diabetes show a stronger γδ T cell response compared to those without this comorbidity. Further studies exploring the relevance of potentially protective γδ T cell responses in melioidosis with diabetes comorbidity are underway.

In conclusion, we demonstrate a role for CX3CR1 and NK cells in survival from acute melioidosis. Our study highlights different patterns in the immune response of people with and without DM comorbidity, implicating a role for CD8^+^ T cells and intact antigen presentation in individuals without DM and humoral immune responses in conjunction with DN and γδ T cells in individuals with DM. Due to some fundamental differences in these two patient groups, stratification of data based on DM status is essential to inform the development of efficient therapeutic interventions and vaccines specifically targeted at this major risk group. Further work studying functional differences including defective cytokine signaling, impaired co‐stimulation, and changes in cellular and systemic metabolism are required to define immune correlates of survival and protection in melioidosis patients with DM.

## Materials and methods

### Ethics statement

Human study protocols were approved by the ethics committees of the Faculty of Tropical Medicine, Mahidol University, of Sunpasitthiprasong Hospital, Ubon Ratchathani and the Oxford Tropical Research Ethics Committee. The study was conducted according to the principles of the Declaration of Helsinki (2008) and the International Conference on Harmonization Good Clinical Practice guidelines. Written informed consent was obtained for all patients enrolled in the study.

### Subjects

Biological samples stored from subjects in longitudinal studies on human melioidosis 24 were selected for this study. Characteristics of patients selected for immunophenotyping are shown in Table 1. Briefly, blood samples were collected from patients over 18 years of age with cultured‐confirmed melioidosis at Sunpasitthiprasong Hospital, Ubon Ratchathani, Thailand at a median of 5 days after admission to hospital (IQR 4–6 days). Twenty‐six percent of patients with acute melioidosis studied on enrolment (week 0) died within 28 days (28DMort). Sixty‐seven percent had a diagnosis of diabetes (defined for the purpose of this study as a past medical history of diabetes and/or a blood glycated haemoglobin (HbA1c) of ≥6.5%) 24. HIV is not a major risk factor for melioidosis in this population 62, and HIV testing was not performed. However, HIV is known to be associated with increased risk of bacterial infections in general 63, and adequately designed and powered studies to quantify the relationship between HIV and melioidosis have not been done. Where possible, follow‐up samples were obtained between weeks 1 and 52 post enrolment. Healthy control subjects and diabetes control subjects were recruited from the blood donation clinic and diabetes outpatient clinic, respectively, at Sunpasitthiprasong Hospital. Control subjects were selected as seronegative controls for melioidosis if their IHA titer was <1:40. Twenty‐eight day survival status was determined using hospital mortality records and contact by telephone.

### Peripheral blood mononuclear cells

Cryopreserved PBMCs isolated as previously described 24 were thawed at 37⁰C and slowly transferred to pre‐warmed R10 media: RPMI 1640 (Sigma, St. Louis, MO, USA) supplemented with 10% heat‐inactivated FCS (Life Technologies, Carlsbad, CA, USA), 1mM Pen/Strep and 2 mM l‐glutamine (both from Sigma). Cells were washed once in R10 and treated with 25 Units Benzonase (Merck Millipore, Billerica, MA, USA) for 30 min at 37⁰C, 5% CO_2_, 95% humidity. After centrifugation, cells were resuspended in R10 and counted using the Scepter cell counter (Merck Millipore) for phenotyping assays or a hemocytometer with trypan blue staining (Sigma) to assess viability for functional assays. For ex vivo phenotyping, cells were immediately subjected to flow cytometry staining. Samples with a viability of less than 20% were excluded from analysis. For intracellular cytokine assays, cells were rested at 3 × 10^6^ cells/mL in R10 at 37⁰C, 5% CO_2_, 95% humidity overnight, followed by counting and culture as described below.

### Intracellular cytokine assay

PBMC were plated at 1 × 10^6^ live cells/well into 96‐well round bottom plates and stimulated with a final concentration of 50 μg/mL soluble antigens derived from heat inactivated (HIA) *BP* (K96243 and clinical isolates 199a and 207a) in the presence of co‐stimulatory molecules αCD28 and αCD49d (1 mg/mL, BD Biosciences, Franklin Lakes, NJ, USA) at a final concentration of 1 μg/mL each. R10 was used as negative control and 5 μg/mL staphylococcal enterotoxin B (Sigma) as positive control. In some experiments, PBMCs were treated with 0.3 μg/mL cyclosporine A (CsA, LKT Labs, St. Paul, MN, USA) in addition to antigenic stimuli and co‐stimulants. In all cases, PBMCs were incubated for 6 h at 37⁰C, 5% CO2, 95% humidity. Brefeldin A (Biolegend, San Diego, CA, USA) was added at a final dilution of 1:1000, 2 h after the addition of stimulants and PBMCs were incubated for further 4 h prior to flow cytometry staining as described below.

### Flow cytometry staining

A MIFlowCyt file (minimum information about a flow cytometry experiment) was created as per Section VI. 4 of “Guidelines for the use of flow cytometry and cell sorting in immunological studies” 64 and recommended by the International Society for Advancement of Cytometry 65. The file contains details of antibodies, reagents, instrument settings, gating strategies, and controls used for flow cytometry experiments and is provided in the supplementary information of this manuscript. PBMCs were resuspended in MACS buffer (Miltenyi Biotec, Bergisch Gladbach, Germany) and incubated for 20 min with near‐infrared live/dead fixable stain (Invitrogen, Carlsbad, CA, USA) and fluorochrome‐conjugated primary human‐specific antibodies in the presence of human FcR blocking reagent (Miltenyi Biotec) at 4⁰C. After washing with MACS buffer, cells were resuspended in IC fixation solution (eBioscience, San Diego, CA, USA) or subjected to intracellular staining. For the latter, cells were fixed with fixation/permeabilization solution (BD Biosciences, eBioscience) for 20 min at 4⁰C, washed with permeabilization buffer (BD Biosciences, eBioscience) followed by incubation with fluorochrome‐conjugated human‐specific antibodies in the presence of FcR blocking reagent. After washing with permeabilization buffer, the samples were resuspended in 1× PBS and acquired on an MACSQuant Analyzer 10 (Miltenyi Biotec) or stored at 4⁰C in the dark for up to 24 h prior to acquisition. Data analysis was performed with FlowJo Version 10 (FlowJo LLC, Ashland, OR, USA) and specific gating strategies can be found in the Supporting Information (MIFlowCyt File). Absolute frequencies of cell populations (live cells per milliliter of blood) were calculated as follows: the number of live PBMCs per milliliter of whole blood was calculated by applying the relative frequency (%) of live cells obtained by FACS to the PBMC yield post isolation. The number of specific cell populations per milliliter of whole blood was then calculated by applying the relative frequency of these cells in the live cell gate to the number of live PBMC per milliliter of whole blood.

### Cytokine detection in serum

Serum levels of IL‐10 and TNF‐α were measured using the Milliplex MAP Human High sensitivity T‐cell panel kit (Merck Millipore) according to the manufacturer's instructions. Serum levels of IL‐15, IL‐18, and IL‐18/IL‐18BPa complex were quantified by ELISA kits according to the manufacturer's instructions (R&D systems, Minneapolis, MN, USA). Results were obtained as absorbance value (OD450) using the Multiskan™ GO Microplate Spectrophotometer (Thermo Scientific, Waltham, MA, USA). Concentrations of cytokines were calculated from standard curves. For the IL‐15 Quantikine ELISA kit, assay diluent was added to a capture antibody pre‐coated ELISA plate followed by incubation with standard or patient's serum for 3 h. Subsequent detection, development, and analysis were performed as described above.

### Statistical analyses

Categorical variables (including sex, risk factors, BC^+^) are displayed as counts and proportions, and were compared using Pearson's Chi Square test. Phenotypic data are presented as box and whiskers blots and cytokine data as median with 95% confidence interval. Statistical differences between control and survived, control and died, or survived and died groups were analyzed by Mann–Whitney *U*‐test for nonparametric data and *t*‐test for parametric data (age and HbA1c) in Graphpad Prism Version 7 (San Diego, CA, USA). To test the association of phenotypic parameters with outcome (28‐day mortality), we performed univariable and multivariable logistic regression adjusting for age, sex, diabetes status, and preexisting renal disease using IBM SPSS Statistics for Windows Version 24 (Armonk, NY, USA). CX3CR1 expression on lymphocytes (CX3CR1^+^ percentage of lymphocytes) and the number of circulating intermediate monocytes (cells per milliliter blood) were analyzed as categorical variables by assigning each subject to one of two groups using the 25% percentile and 75% percentile of the survived cohort as cut‐off, respectively. In all analyses, a *p*‐value (two‐tailed) of ≤0.05 was considered statistically significant.

## Author contributions

B.K. and S.D. conceptualized and designed the study. D.L., P.T., N.D., and S.D. were associated with patient recruitment and clinical study oversight. B.K., P.C., M.S., K.J., F.C., S.C., and S.D. were associated with collection and assembly of data. B.K., P.C., M.S., F.C., D.L., P.K., and S.D. were associated with data analysis and interpretation. B.K., P.C, D.L., and S.D. performed statistical analysis. B.K., D.L., N.D., P.K., and S.D. supervised the study. P.C., F.C., N.D., and S.D. were associated with funding acquisition. B.K., P.C., and S.D. wrote the manuscript. All the authors were associated with review/editing and final approval of manuscript.

## Conflict of interest

The authors declare no commercial or financial conflict of interest.

**Abbreviations**
*BP*: *Burkholderia pseudomallei*
DM: diabetes mellitus
GzmB: granzyme B
IHA: indirect hemagglutination assay
pDC: plasmacytoid dendritic cells
T2D: type 2 diabetes

## Supporting information

Supporting Information
