# Supplementary material for: Diabetes alters immune response patterns to acute melioidosis in humans
Source: Eur J Immunol. 2019 May 8;49(7):1092–106. doi: 10.1002/eji.201848037 (PMC6618312; doi:10.1002/eji.201848037)
Supplement: Supplementary file 1 — Supporting Information [file EJI-49-1092-s001.pdf]

European Journal of Immunology

Supporting Information

for

DOI 10.1002/eji.201848037

Barbara Kronsteiner, Panjaporn Chaichana, Manutsanun Sumonwiriya, Kemajitra Jenjaroen, Fazle Rabbi Chowdhury, Suchintana Chumseng, Prapit Teparrukkul, Direk Limmathurotsakul, Nicholas P.J. Day, Paul Klenerman and Susanna J. Dunachie

Diabetes alters immune response patterns to acute melioidosis in humans

© 2019 The Authors. *European Journal of Immunology* published by WILEY-VCH Verlag GmbH & Co. KGaA, Weinheim.

SUPPORTING INFORMATION

Supplementary Figure 1

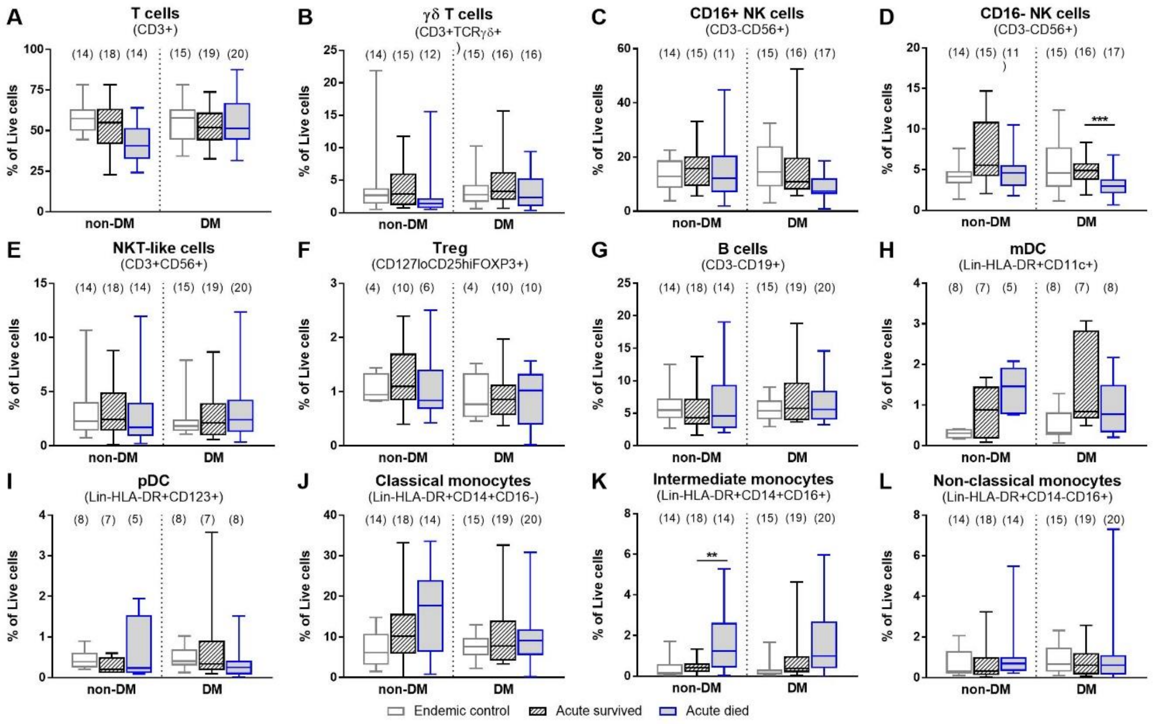

Supplementary Figure 1. Circulating immune cell subsets during acute melioidosis. Circulating immune cell subsets during acute melioidosis. The relative frequency (% of all live cells) of (A) T cells, (B) γδ T cells, (C) CD16+ NK cells, (D) CD16- NK cells, (E) NKT-like cells, (F) regulatory T cells (Treg), (G) B cells, (H) myeloid (m) and (I) plasmacytoid (p) dendritic cells (DC) as well as (J) classical, (K) intermediate, (L) and non-classical monocytes was determined by multicolour flow cytometry of PBMC from acute melioidosis patients with (DM) and without diabetes (non-DM), who survived (black, striped) and died (blue, filled), as well as endemic controls (grey). Six experiments with 3-4 biological replicates/group were performed. Total number of biological replicates per group are given in brackets on top of blots. Data is presented in box and whiskers blots Mann-Whitney-U test was performed on survived versus died groups and significant differences are depicted as **p≤0.01, ***p≤0.001. The

gating strategy used is described and shown in detail in the MIFlowCyt file provided as supplementary information.

Supplementary Figure 2

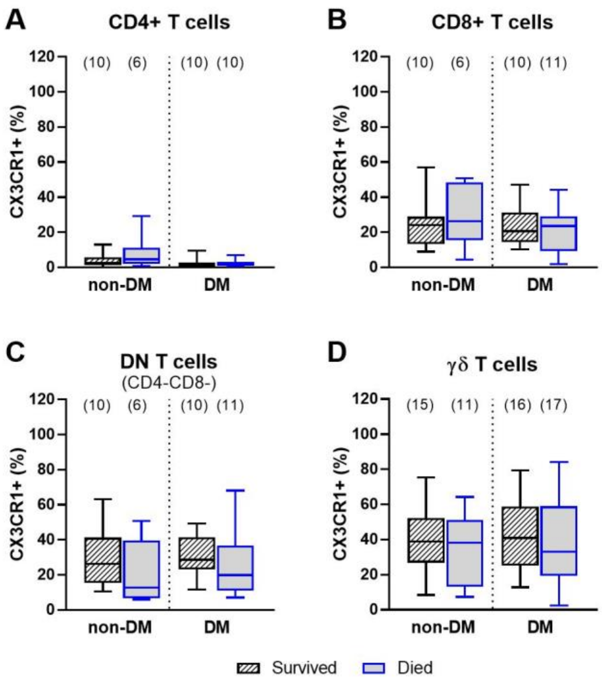

Supplementary Figure 2. CX3CR1 expression on T cells. CX3CR1 expression was assessed on (A) $CD4^+$, (B) $CD8^+$, (C) $CD4^-CD8^-$ double negative (DN) T cells and (D) γδ T cells in survived (black, striped) and died (blue, filled) acute melioidosis patients with (DM) and without (non-DM) diabetes. Four experiments with 3-4 biological replicates/group were performed. Total number of biological replicates per group are given in brackets on top of blots. Data is presented in box and whiskers blots. Mann-Whitney-U test was performed on survived versus died groups and no significant differences were detected. The gating strategy used is described and shown in detail in the MIFlowCyt file provided as supporting information.

Supplementary Figure 3

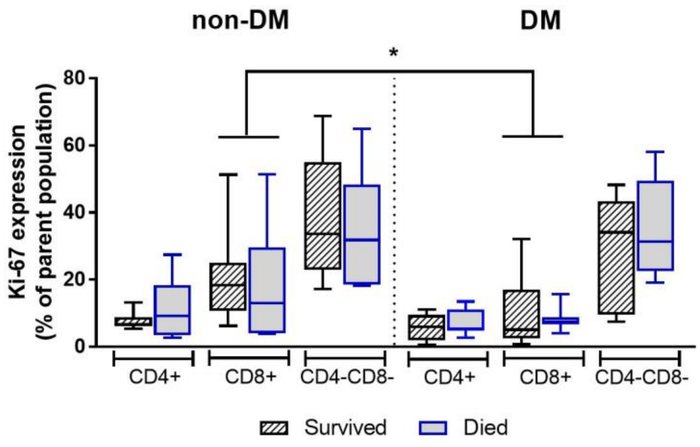

Supplementary Figure 3. T cell proliferation in acute melioidosis. The frequency of Ki-67 expressing, proliferating cells was determined within CD4+, CD8+ and CD4-CD8- T cell subsets of survived (black, striped) and died (blue, filled) acute melioidosis patients without (non-DM, n=6-7/group) and with diabetes (DM, n=7-8/group). Data is presented in box and whiskers blots. Mann-Whitney-U test was performed to compare survived versus died groups and acute melioidosis patients with and without DM. Significant differences are depicted as $*p \leq 0.05$. The gating strategy used is described and shown in detail in the MIFlowCyt file provided as supporting information.

Supplementary Table 1. Circulating immune cell phenotype of melioidosis and endemic control cohorts.

		non-DM										DM									
		Control			Survived			Died			*P* value	Control			Survived			Died			*P* value
**Cell population**	**Unit**	median	range	n	median	range	n	median	range	n	(Surv vs. Died)	median	range	n	median	range	n	median	range	n	(Surv vs. Died)
T cells	x10^5 live cells/ml blood	**8.3**	3.4-24.3	14	**6.9**	2.3-21.0	18	**3.9**	0.9-16.0	14	<0.01*	**7.4**	3.6-11.8	15	**6.7**	1.9-16.8	19	**5.2**	2.2-20.9	20	0.40
CD4+ T cells	x10^5 live cells/ml blood	**4.8**	1.4-12.8	14	**4.7**	1.8-8.6	13	**3.6**	0.5-6.1	9	0.82	**3.5**	1.7-7.2	15	**3.5**	1.7-9.4	13	**2.7**	1.9-13.0	13	0.36
CD8+ T cells	x10^5 live cells/ml blood	**1.9**	0.8-6.4	14	**2.1**	0.7-7.5	13	**0.8**	0.3-5.5	9	<0.01*	**1.9**	0.7-3.8	15	**1.2**	0.2-3.4	13	**1.2**	0.6-5.2	14	0.69
CD4-CD8- T cells	x10^4 live cells/ml blood	**6.0**	1.1-24.3	14	**8.4**	2.5-35.5	10	**7.1**	0.9-41.5	7	>0.99	**6.2**	2.5-16.3	15	**10.2**	2.5-24.1	10	**3.1**	0.7-13.5	11	0.02*
γδ T cells	x10^4 live cells/ml blood	**4.6**	0.7-23.7	14	**4.3**	0.9-31.5	15	**1.6**	0.4-41.8	12	0.11	**3**	0.9-10.9	15	**4.8**	1.0-22.0	16	**1.7**	0.4-12.0	17	0.06
Treg	x10^4 live cells/ml blood	**2.0**	1.3-3.3	4	**1.9**	0.3-6.6	10	**1.3**	0.2-2.7	6	0.43	**1**	0.5-2.3	4	**1.2**	0.3-3.0	10	**1.3**	0.02-3.3	10	0.63
B cells	x10^4 live cells/ml blood	**8.2**	3.6-39.0	14	**6.7**	2.0-21.7	18	**4.8**	2.2-18.7	14	0.11	**6.6**	3.3-15.4	15	**7.2**	3.0-31.1	19	**6.8**	1.3-28.7	20	0.69
CD16+ NK cells	x10^4 live cells/ml blood	**11.4**	2.4-27.7	14	**10.0**	0.2-36.6	15	**7.2**	0.8-28.8	11	0.24	**10.7**	2.2-28.9	15	**8.1**	2.1-69.8	16	**5.7**	0.5-14.7	17	1.46
CD16- NK cells	x10^4 live cells/ml blood	**6.3**	2.1-10.6	14	**9.5**	4.5-18.3	15	**3.9**	1.1-11.9	11	<0.01*	**5.2**	1.3-15.2	15	**7.2**	1.4-19.1	16	**2.6**	1.4-13.0	17	<0.01*
NKT-like cells	x10^4 live cells/ml blood	**2.6**	1.0-8.6	14	**3.9**	0.2-20.5	18	**1.9**	0.2-32.1	14	0.07	**2.1**	1.1-10.7	15	**2.7**	0.8-12.2	19	**2.8**	0.3-16.9	20	0.75
total Monocytes	x10^5 live cells/ml blood	**0.9**	0.2-2.6	14	**2.3**	0.005-5.0	18	**1.5**	0.2-5.0	14	0.96	**1.0**	0.5-2.0	15	**1.3**	0.3-3.9	19	**1.2**	0.03-4.4	20	0.69
classical Monocytes	x10^5 live cells/ml blood	**0.8**	0.2-2.5	14	**2.1**	0.003-4.6	18	**1.7**	0.2-4.8	14	0.96	**0.9**	0.4-1.5	15	**1.0**	0.3-3.3	19	**0.9**	0.02-3.9	20	0.44
HLA-DR on classical M	MFI	**10367**	8610-10901	14	**9183**	5927-14241	18	**5326**	3629-8207	14	0.03*	**13065**	9709-16087	15	**8570**	6346-10969	19	**7011**	3712-11218	20	0.18
intermediate Monocytes	x10^3 live cells/ml blood	**2.2**	0.2-27.3	14	**5.2**	0.04-21.0	18	**12.2**	0.1-38.9	14	0.15	**1.6**	0.6-17.7	15	**3.5**	0.8-50.7	19	**10.3**	0.2-64.8	20	0.14
non classical Monocytes	x10^3 live cells/ml blood	**5.1**	1.5-32.6	14	**5.7**	0.1-66.4	18	**7.4**	0.7-34.2	14	0.81	**7.7**	1.5-38.8	15	**8.5**	0.8-32.1	19	**5.5**	0.1-55.9	20	0.53
mDC	x10^3 live cells/ml blood	**5.4**	2.6-9.6	8	**20.4**	0.6-38.9	7	**11.9**	5.9-47.7	5	0.88	**3.8**	1.0-17.8	8	**10.8**	5.9-42.6	7	**13.5**	1.5-29.7	8	0.61
pDC	x10^3 live cells/ml blood	**7.4**	2.1-15.4	8	**7.8**	0.8-11.7	7	**2.0**	0.7-30.7	5	0.755	**5.2**	1.9-19.0	8	**11.3**	1.1-27.7	7	**4.1**	0.05-20.8	8	0.28
HLA-DR on pDC	MFI	**15429**	12385-18121	8	**13976**	12509-14379	7	**6789**	3656-10007	5	0.02*	**10994**	9209-17096	8	**5996**	2847-8917	7	**7557**	3757-10858	8	0.54
CX3CR1 expression	% of lymphocytes	**31.8**	21.1-37.9	14	**34.8**	23.3-44.9	18	**22.8**	9.7-30.2	14	0.02*	**32.9**	22.9-37.2	15	**28.4**	19.8-38.6	19	**23.7**	15.1-26.4	20	0.21
CX3CR1+ lymphocytes	x10^5 live cells/ml blood	**3.5**	1.2-6.5	14	**3.7**	0.9-8.8	18	**1.0**	0.1-8.0	14	<0.01*	**2.7**	1.6-6.1	15	**2.3**	0.7-10.7	19	**1.5**	0.1-6.3	20	0.09

DM=diabetes mellitus, Treg= regulatory T cell, NK=natural killer, M=monocytes, mDC=myeloid dendritic cell, pDC=plasmacytoid dendritic cell, MFI=median fluorescence intensity

Mann-Whitney U test was performed to compare survived with died patients, *$p \leq 0.05$

Supplementary Table 2. CX3CR1 and GzmB expression on NK cells from acute melioidosis patients.

		non-DM							DM						
		Survived			Died				Survived			Died			
**NK cell subset**	**MFI**	median	range	n	median	range	n	p value	median	range	n	median	range	n	p value
CX3CR1+CD16+	CX3CR1	754	712-1012	5	490	288-767	4	0.11	719	646-950	5	723	467-1151	5	>0.99
CX3CR1+CD16-	CX3CR1	618	402-976	5	393	284-548	4	0.06	551	466-774	5	520	422-861	5	0.55
GzmB+CD16+	GzmB	3967	3107-4272	5	2454	1899-2963	4	0.02*	3800	2193-4176	5	2563	2099-3649	5	0.22
GzmB+CD16-	GzmB	3604	3083-4049	5	2444	2168-2609	4	0.02*	3400	2173-3902	5	3028	1979-3701	5	0.69

DM=diabetes mellitus, NK=natural killer (CD3-CD19-CD14-CD56+), MFI=median fluorescence intensity, GzmB=Granzyme B

Mann-Whitney U test was performed to compare survived with died patients, *p≤0.05

MIFlowCyt file

1. Experiment overview

1.1. Purpose

The aim of this study was to identify immune correlates of survival in acute human melioidosis and possible differences based on diabetes co-morbidity by analysing circulating immune cell populations and functional properties thereof.

1.2. Keywords

Melioidosis, *B. pseudomallei*, correlates of survival, PBMC, immunophenotyping, re-call response, IFN-γ production

1.3. Experiment variables

1.3.1. *Ex vivo* phenotyping:

Acute melioidosis patients (M) without (NDM) and with (DM) diabetes (HbA1c<7%), who survived (S) or died (D) from disease (based on 28 day mortality). Endemic controls (C) without and with diabetes.

- M-NDMS: n=7-18
- M-NDMD: n=5-14
- M-DMS: n=7-19
- M-DMD: n=8-20
- C-NDM: n=4-14
- C-DM: n=4-15

1.3.2. Intracellular cytokine (ICCK) assay:

Survivors of acute melioidosis with and without diabetes (HbA1c<7%) 1-52 weeks post enrolment. n=5-11

Stimulants:

- soluble *B. pseudomallei* antigens+ αCD28/αCD49d±DMSO or cyclosporine A (CsA)
- R10 (RPMI 1640, 10% FBS, 1mM Pen/Strep, 2mM L-Glut) + αCD28/αCD49d±DMSO or cyclosporine A (CsA)
- staphylococcal enterotoxin B (SEB) + αCD28/αCD49d±DMSO or cyclosporine A (CsA)

1.4. Organization

1.4.1. Name: Tropical Immunology Lab, NDM Tropical Medicine, University of Oxford, Oxford, United Kingdom
Mahidol Oxford Research Unit, University of Oxford and Mahidol University, Bangkok, Thailand

1.4.2. Address: Peter Medawar Institute for Pathogen Research, South Parks Road, Oxford, OX1 3SY, United Kingdom

1.5. Contacts

Name 1: Susanna Dunachie (PI)
e-Mail 1: Susie.dunachie@ndm.ox.ac.uk

Name 2: Barbara Kronsteiner-Dobramysl (PostDoc)
e-Mail 2: Barbara.kronsteiner-dobramysl@ndm.ox.ac.uk

1.6. Date

Experiments were performed between March 2016 and July 2017.

1.7. Conclusions

The study of circulating immune cells showed differences in immune pathways important for survival from acute melioidosis in patients with and without diabetes.

1.8. Quality control measures

The performance of the flow cytometer was analysed daily by running quality control beads according to the manufacturer's recommendations (for details see section 2.4.1). Appropriate fluorescence minus one (FMO) controls were used as quality control for software computed compensation and to set gate boundaries.

2. Sample/Specimen Material Description

2.1. Biological Samples

2.1.1. Biological sample description:
PBMC (groups see under 1.3.)

2.1.2. Biological Sample Source Description:

Whole blood

2.1.3. Biological Sample Source Organism Description:

Taxonomy: Homo Sapiens
Age: 23-79
Gender: male and female
Phenotype: not applicable
Genotype: not applicable
Treatment: antibiotics to treat acute melioidosis and in case of patients with diabetes insulin and/or oral anti-diabetic drugs
Groups: see sections 1.3.1 and 1.3.2

2.2. Sample Characteristics

Expected sample characteristics are the main circulating immune cell subsets (T cells, B cells, NK cells, NKT cells, monocytes, dendritic cells) typically found in peripheral blood. In case of ICCK the expected sample characteristic is IFN-γ secretion upon stimulation with cognate antigen.

2.3. Sample treatment Description

- For *ex vivo* phenotyping, cells were used immediately after thawing.

- For ICCK assays, cells were rested overnight and then cultured in the presence of bacterial antigen and co-stimulants for 6 hours. Media served as negative control and staphylococcal

enterotoxin B as positive control for cytokine secretion. In some cases cells were concomitantly treated with cyclosporine A or vehicle control (DMSO). Brefeldin A was added for the last 4 hours of incubation to inhibit cytokine transport from the endoplasmatic reticulum to the Golgi apparatus.

- Multicolour flow cytometry staining:
  PBMC were resuspended in MACS buffer and incubated for 20min with near-infrared live/dead fixable stain and fluorochrome-conjugated primary human-specific antibodies in the presence of human FcR blocking reagent at 4°C. After washing with MACS buffer, cells were resuspended in IC fixation solution or subjected to intracellular staining. For the latter, cells were fixed with fixation/permeabilization solution for 20 min at 4°C , washed with permeabilization buffer followed by incubation with fluorochrome-conjugated human-specific antibodies in the presence of FcR blocking reagent. After washing with permeabilization buffer the samples were resuspended in 1xPBS and acquired on a MACSQuant Analyzer 10 or stored at 4°C in the dark for up to 24 hours prior to acquisition. Data analysis was performed with FlowJo Version 10 (FlowJo, LLC, Oregon, USA) and specific gating strategies can be found in the Supplementary information.

2.4. Fluorescence Reagent Description

2.4.1. Reagents and Antibodies

Reagent Name	Catalogue No	Manufacturer
Anti-mouse compensation beads	552843	BD Biosciences
Anti-rat/anti-hamster IgGk/Negative control compensation particles set	552845	BD Biosciences
ArC™ Amine Reactive Compensation Bead Kit	A10346	Life Technologies
Brefeldin A Solution 1000x	420601	Biolegend
Cyclosporin A	C9611-10 mg	LKT Labs
DMSO	D2650-100ML	Sigma
FBS heat inactivated	10500064	Life Technologies
Fixation/Permeabilization Kit	554714	BD Biosciences
FOXP3 Transcription Factor Staining Buffer Set	00-5523-00	eBioscience
human Fc Blocking reagent	130-059-901	Miltenyi Biotec
IC Fixation Buffer	00-8222-49	Life Technologies
L-Glutamine	G7513-100ML	Sigma
Live/Dead Fixable Near IR Dead Cell Stain Kit	L10119	Life Technologies
MACS Comp Bead Kit, anti-REA	130-104-693	Miltenyi Biotec
MACSQuant Calibration Beads	130-093-607	Miltenyi Biotec
MACSQuant Running Buffer	130-092-747	Miltenyi Biotec
Pen-Strep	P0781-100ml	Sigma
Purified Mouse Anti-Human CD28	340975	BD Biosciences
Purified Mouse Anti-Human CD49d	340976	BD Biosciences
RPMI 1640 sodium bicarbonate, without L-Glut	R0883-500ml	Sigma
Staphylococcal enterotoxin B	S4881-1MG	Sigma

Marker	Fluorchrome/ Reporter	Channel	Clone	Target species	Host species	Isotype	Cat No	Manufacturer	Stock conc. (µg/ml)	Final conc. (µg/ml)	Optimal dilution	Buffer system	Characteristic measured
Caspase 3 (activated)	PE	B2	C92-605	human	rabbit	IgG	550821	BD	NA	NA	8	BD Cytofix/Cytoperm	Apoptosis, Intracellular protein
CCR2	BV510	V2	K036C2	human	mouse	IgG2a, k	357218	Biolegend	100	2.5	40	MACS buffer	Cell surface protein
CD11c	BV510	V2	3.9	human	mouse	IgG1, k	301634	Biolegend	200	4.0	50	MACS buffer	Cell surface protein
CD123	eFluor450	V1	6H6	human	mouse	IgG1, k	48-1239-41	eBioscience	50	0.6	80	MACS buffer	Cell surface protein
CD127	AF647	R1	A019D5	human	mouse	IgG1, k	351317	Biolegend	100	1.0	100	MACS buffer	Cell surface protein
CD14	PerCP	B3	HCD14	human	mouse	IgG1, k	325632	Biolegend	300	1.5	200	MACS buffer	Cell surface protein
CD16	Pacific Blue	V1	3G8	human	mouse	IgG1, k	302021	Biolegend	500	5.0	100	MACS buffer	Cell surface protein
CD161	PE-Cy7	B4	HP-3G10	human	mouse	IgG1, k	339918	Biolegend	100	2.5	40	MACS buffer	Cell surface protein
CD19	FITC	B1	HIB19	human	mouse	IgG1, k	302206	Biolegend	400	2.0	200	MACS buffer	Cell surface protein
CD25	PE	B2	M-A251	human	mouse	IgG1, k	356104	Biolegend	100	2.0	50	MACS buffer	Cell surface protein
CD3	PE-Cy5	B3	UCHT1	human	mouse	IgG1, k	300410	Biolegend	25	0.1	200	MACS buffer	Cell surface protein
CD3	PerCP	B3	UCHT1	human	mouse	IgG1, k	300428	Biolegend	200	2.0	100	MACS buffer	Cell surface protein
CD3	BV510	V2	UCHT1	human	mouse	IgG1, k	300448	Biolegend	100	1.0	100	MACS buffer	Cell surface protein
CD4	V450	V1	L200	human	mouse	IgG1, k	560811	BD	NA	NA	160	MACS buffer	Cell surface protein
CD56	APC	R1	B159	human	mouse	IgG1, k	555518	BD	NA	NA	10	MACS buffer	Cell surface protein
CD8α	BV510	V2	RPA-T8	human	mouse	IgG1, k	301048	Biolegend	100	0.2	600	MACS buffer	Cell surface protein
CD86	APC	R1	IT2.2	human	mouse	IgG2b	305412	Biolegend	200	4.0	50	MACS buffer	Cell surface protein
CTLA-4 (CD152)	PE-Cy7	B4	L3D10	human	mouse	IgG1, k	349913	Biolegend	150	1.9	80	eBio FOXP3 Fix/Perm	Cell surface protein
CX3CR1	PE	B2	2A9-1	human	rat	IgG2b	341604	Biolegend	150	1.0	150	MACS buffer	Cell surface protein
FOXP3	PE-Cy7	B4	236A/E7	human	mouse	IgG1, k	25-4777-41	eBioscience	25	1.3	20	eBio FOXP3 Fix/Perm	Intracellular protein
Granzyme B	AF647	R1	GB11	human	mouse	IgG1, k	515405	Biolegend	20	0.3	80	eBio FOXP3 Fix/Perm	Intracellular protein
HLA-DR	FITC	B1	L243	human	mouse	IgG2a, k	307604	Biolegend	400	13.3	30	MACS buffer	Cell surface protein
HLA-DR, DP, DQ	PE Vio-770	B4	REA332	human	rhuman	IgG1	130-104-828	Miltenyi	na	na	100	MACS buffer	Cell surface protein
IFN-γ	PE	B2	4S.B3	human	mouse	IgG1, k	502508	Biolegend	10	0.2	50	BD Cytofix/Cytoperm	Intracellular protein
IFN-γ	PE-Cy7	B4	4S.B3	human	mouse	IgG1, k	502528	Biolegend	50	1.3	40	BD Cytofix/Cytoperm	Intracellular protein
Ki-67	APC	R1	Ki-67	human	mouse	IgG1, k	350513	Biolegend	50	0.4	120	eBio FOXP3 Fix/Perm	Cell cycle
Lineage (CD3, CD14, CD19, CD20, CD56)	FITC	B1	UCHT1, HCD14, HIB19, 2H7, HCD56	human	mouse	IgG1, IgG2b	348701	Biolegend	NA	NA	10	MACS buffer	Cell surface protein
Lineage (CD3, CD19, CD20, CD56)	APC	R1	UCHT1, HIB19, 2H7, 5.1H11	human	mouse	IgG1, IgG2b	363601	Biolegend	NA	NA	20	MACS buffer	Cell surface protein
PD-1 (CD279)	PE	B2	MIH4	human	mouse	IgG1, k	557946	BD	NA	NA	15	MACS buffer	Cell surface protein
TCRγδ	FITC	B1	B1	human	mouse	IgG1, k	331208	Biolegend	600	24.0	25	MACS buffer	Cell surface protein
TCRγδ	APC	R1	B1	human	mouse	IgG1, k	331212	Biolegend	200	6.7	30	MACS buffer	Cell surface protein
Fixable Near IR Dead Cell Stain Kit	Near-infrared	R2	NA	NA	NA	NA	L10119	Life Technologies	NA	NA	1000	MACS buffer	Cell death

2.4.2. Antibody panels used in *ex vivo* phenotyping experiments

Panel 1: Monocytes and Lymphocyte activation/cytotoxicity					
**Marker/Characteristic**	**Fluorochrome /Reporter**	**Stock conc. (ug/ml)**	**Final conc. (ug/ml)**	**Dilution**	**Details**
Viability	Near IR	na	na	1000	Dead cells
Lineage (CD3, 19, 20, 56)	APC	na	na	20	T, B, NK cells
HLA-DR	FITC	400	13.33	30	Activation, Ag presentation
CD14	PerCP	300	1.50	200	Monocytes
CD16	Pacific Blue	500	5.00	100	FcγRIII
CCR2	BV510	100	2.50	40	Monocyte chemotaxis
CX3CR1	PE	150	1.00	150	Homing, cytotoxicity
FcR Blocking		na	na	10	
MACS Running Buffer					

Panel 2: Apoptosis and Lymphocyte subsets					
**Marker/Characteristic**	**Fluorochrome /Reporter**	**Stock conc. (ug/ml)**	**Final conc. (ug/ml)**	**Dilution**	**Details**
Viability	Near IR	na	na	1000	Dead cells
CD3	BV510	100	1.00	100	Pan T cell
CD56	APC	na	na	10	NK cells
CD19	FITC	400	2.00	200	B cells
CD14	PerCP	300	1.50	200	Monocytes
CD16	Pacific Blue	500	5.00	100	FcγRIII
FcR Blocking		na	na	10	
MACS Running Buffer					
Caspase 3 (activated)	PE	na	na	8	Apoptosis
FcR Blocking		na	na	10	
BD Perm/Wash buffer					

Panel 3: Regulatory T cells (Treg)					
**Marker/Characteristic**	**Fluorochrome /Reporter**	**Stock conc. (ug/ml)**	**Final conc. (ug/ml)**	**Dilution**	**Details**
Viability	Near IR	na	na	1000	Dead cells
CD3	BV510	100	1.00	100	Pan T cell
CD4	V450	na	na	160	T helper cell
CD25	PE	100	2.00	50	Activation
CD127	AF647	100	1.00	100	IL-7Rα
HLA-DR	FITC	400	13.33	30	Activation, Ag presentation
FcR Blocking		na	na	10	
MACS Running Buffer					
FOXP3	PE-Cy7	25	1.25	20	Transcription factor
FcR Blocking		na	na	10	
eBioscience Perm/Wash buffer					

Panel 4: Cytotoxic lymphocytes					
**Marker/Characteristic**	**Fluorochrome /Reporter**	**Stock conc. (ug/ml)**	**Final conc. (ug/ml)**	**Dilution**	**Details**
Viability	Near IR	na	na	1000	Dead cells
CD3	PE-Cy5	25	0.13	200	Pan T cells
CD8	BV510	100	0.17	600	CD8+ T cells
CD4	V450	na	na	160	CD4+ T cells
TCRγδ	FITC	600	24.00	25	γδ T cells
CD161	PE-Cy7	100	2.50	40	NK, ILC, unconventional T
CX3CR1	PE	150	1.00	150	Homing, cytotoxicity
FcR Blocking		na	na	10	
MACS Running Buffer					
Granzyme B	AF647	20	0.25	80	Serine protease
FcR Blocking		na	na	10	
eBioscience Perm/Wash buffer					

Panel 5: Exhaustion and proliferation					
**Marker/Characteristic**	**Fluorochrome /Reporter**	**Stock conc. (ug/ml)**	**Final conc. (ug/ml)**	**Dilution**	**Details**
Viability	Near IR	na	na	1000	Dead cells
CD3	PerCP	200	2.00	100	Pan T cells
CD4	V450	na	na	160	T helper cells
CD8	BV510	100	0.17	600	Cytotoxic T cells
HLA-DR	FITC	400	13.33	30	Activation, Ag presentation
PD-1	PE	na	na	15	Checkpoint inhibitor
FcR Blocking		na	na	10	
MACS Running Buffer					
CTLA-4	PE-Cy7	150	1.88	80	Co-stimulation
Ki-67	APC	50	0.42	120	Proliferation
FcR Blocking		na	na	10	
eBioscience Perm/Wash buffer					

Panel 6: Dendritic cells					
**Marker/Characteristic**	**Fluorochrome /Reporter**	**Stock conc. (ug/ml)**	**Final conc. (ug/ml)**	**Dilution**	**Details**
Viability	Near IR	na	na	1000	Dead cells
Lin (CD3, 19, 20, 56, 14)	FITC	na	na	10	T, B, NK and Monocytes
HLA-DR, DP, DQ	PE-Vio770	na	na	100	Activation, Ag presentation
CD11c	BV510	200	4.00	50	mDC
CD123	eFluor450	50	0.63	80	pDC
CD86	APC	200	4.00	50	Co-stimulation
FcR Blocking		na	na	10	
MACS Running Buffer					

2.4.3. Antibody panels used in ICCK assays

ICCK Version 1					
**Marker/Characteristic**	**Fluorochrome /Reporter**	**Stock conc. (ug/ml)**	**Final conc. (ug/ml)**	**Dilution**	**Details**
Viability	Near IR	na	na	1000	Dead cells
CD3	PerCP	200	2.00	100	Pan T cells
CD4	V450	na	na	160	CD4+ T cells
CD8	BV510	100	0.17	600	CD8+ T cells
TCRγδ	APC	200	5.00	40	γδ T cells
FcR Blocking		na	na	10	
MACS Running Buffer					
IFN-γ	PE	10	0.25	40	
FcR Blocking		na	na	10	
BD Perm/Wash buffer					

ICCK Version 2					
**Marker/Characteristic**	**Fluorochrome /Reporter**	**Stock conc. (ug/ml)**	**Final conc. (ug/ml)**	**Dilution**	**Details**
Viability	Near IR	na	na	1000	Dead cells
CD3	PE-Cy5	25	0.13	200	Pan T cells
CD4	V450	na	na	160	CD4+ T cells
CD8	BV510	100	0.17	600	CD8+ T cells
TCRγδ	FITC	600	24.00	25	γδ T cells
FcR Blocking		na	na	10	
MACS Running Buffer					
IFN-γ	PE-Cy7	50	1.25	40	
FcR Blocking		na	na	10	
BD Perm/Wash buffer					

2.4.4. Compensation

Compensation was performed using beads (see details in 2.4.1), using single stains for each antibody and one unstained sample. Samples were acquired uncompensated and compensation was later computed in FlowJo V10. FMO controls were used to check accurate compensation.

3. Instrument Details

3.1. Instrument Manufacturer and Model

MACSQuant Analyzer 10 (Miltenyi Biotec)

Optics			
**Laser excitation**	405 nm, 40 mW diode<br>488 nm, 30 mW DPSS (diode pumped solid state)<br>638 nm, 20 mW diode		
**Emission detectors**	**Channel**	**Filter**	**Dye**
	FSC	488/10 nm	Size
	SSC	488/10 nm	Granularity
	V1	450/50 nm	VioBlue®
	V2	525/50 nm	VioGreen™
	B1	525/50 nm	FITC, GFP
	B2	585/40 nm	PE
	B3	655–730 nm	PerCP, PE-Cy™5.5,<br>PerCP-Vio700
	B4	750 nm LP	PE-Vio770™
	R1	655–730 nm	APC
	R2	750 nm LP	APC-Vio770
**Optical alignment**	Fixed tree-like configuration, no user adjustments needed		
**Fluorescence sensitivity and resolution**	MESFs (CV <5%):<br>FITC <200<br>PE <100<br>APC <150		
**Flow cell dimensions**	200×250 µm		
**Fluorescence detectors**	Optimized with spectrally matched PMTs for all channels		

3.2. Instrument Configurations and Settings

The instrument has not been altered

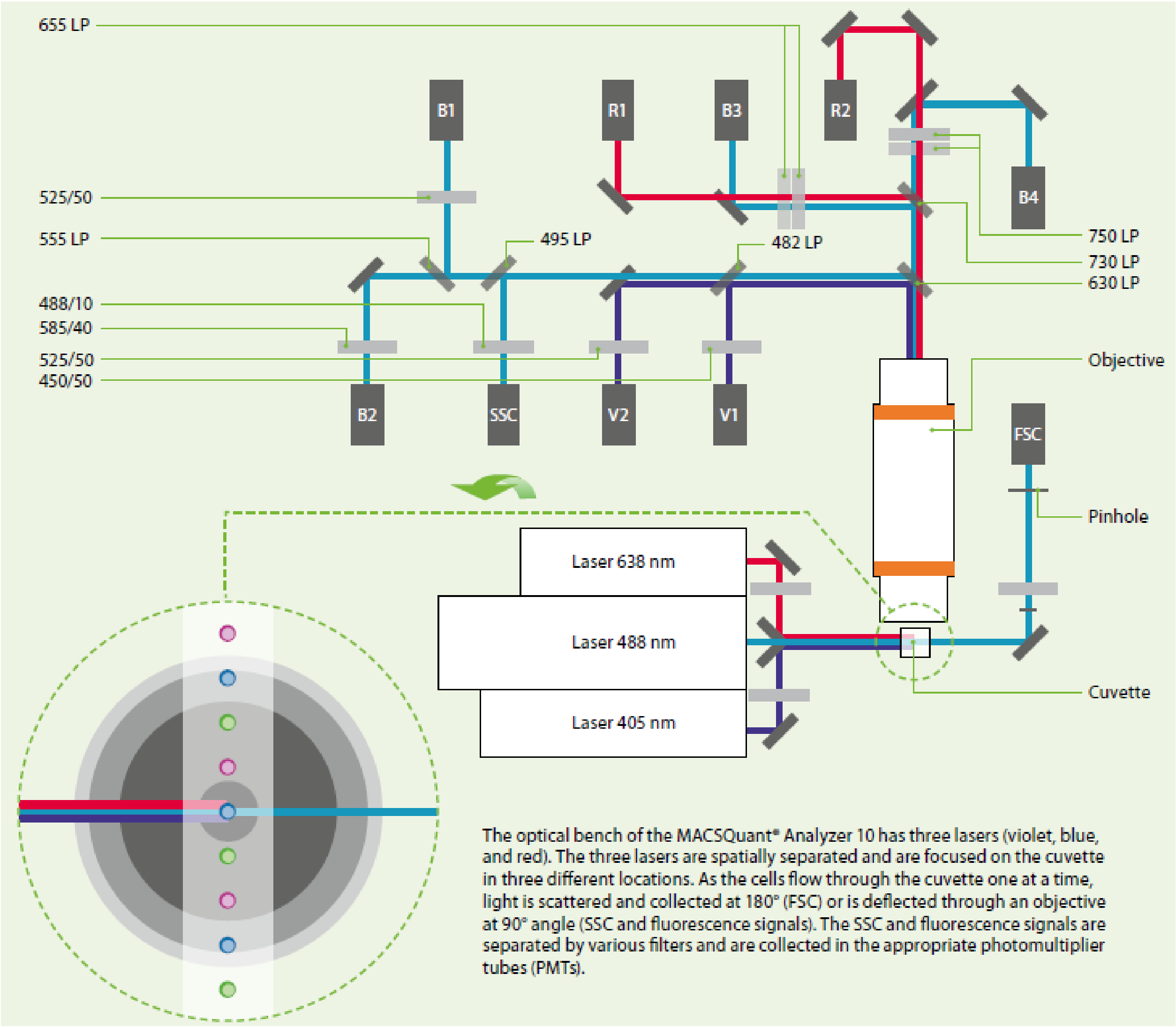

The optical bench of the MACSQuant® Analyzer 10 has three lasers (violet, blue, and red). The three lasers are spatially separated and are focused on the cuvette in three different locations. As the cells flow through the cuvette one at a time, light is scattered and collected at 180° (FSC) or is deflected through an objective at 90° angle (SSC and fluorescence signals). The SSC and fluorescence signals are separated by various filters and are collected in the appropriate photomultiplier tubes (PMTs).

Laser	Channel	Filter (nm)	Dye or parameter
Violet 405 nm	V1	450/50	CFP, VioBlue
	V2	525/50	Pacific Orange™, VioGreen
Blue 488 nm	B1	525/50	GFP, FITC
	B2	585/40	PE
	B3	655-730	PI, PerCP, PE-Cy™5.5, PerCP-Vio700, PE-Vio615, ECD, PE-CF594, PE/Dazzle™ 594, PE-eFluor® 610
	B4	750 LP	PE-Cy7, PE-Vio770
Red 635 nm	R1	655-730	APC
	R2	750 LP	APC-Cy7, APC-Vio770
Blue 488 nm	FSC	488/10	Size
	SSC	488/10	Granularity

Bead calibration of the instrument was performed daily and thereafter the voltage settings were not altered for experiments.

4. Data Analysis Details

4.1. List-mode Data Files

FSC files can be obtained by contacting Prof. Susanna Dunachie (see contact details above) after this work has undergone peer-review and publication.

4.2. Compensation

The compensation matrices shown below are representative examples.

	V1-A :: Pacific Blue	V2-A :: BV510	B1-A :: FITC	B2-A :: PE	B4-A :: PE-Cy7	R1-A :: APC	R2-A :: NiR
V1-A :: Pacific Blue	100.00	26.12	0.02	0.01	-0.01	-0.02	-0.07
V2-A :: BV510	12.38	100.00	0.10	0.02	0.00	0.00	-0.02
B1-A :: FITC	-0.01	5.34	100.00	8.34	0.17	-0.01	-0.20
B2-A :: PE	-0.02	0.46	1.62	100.00	2.34	0.04	-0.02
B4-A :: PE-Cy7	-0.03	0.01	0.12	1.28	100.00	0.31	7.58
R1-A :: APC	-0.02	-0.02	0.01	0.00	0.09	100.00	13.49
R2-A :: NiR	-0.40	-0.10	0.19	0.12	3.61	6.13	100.00

	V1-A :: V450	V2-A :: BV510	B1-A :: FITC	B2-A :: PE	B3-A :: PE-Cy5	B4-A :: PE-Cy7	R1-A :: AF647	R2-A :: NiR
V1-A :: V450	100.00	26.12	0.03	0.01	0.02	-0.01	-0.02	-0.07
V2-A :: BV510	13.11	100.00	0.30	0.02	0.14	0.00	0.06	0.01
B1-A :: FITC	-0.01	3.50	100.00	7.90	1.77	0.17	-0.01	-0.20
B2-A :: PE	0.06	0.50	1.77	100.00	21.44	2.32	0.03	0.13
B3-A :: PE-Cy5	0.04	0.00	0.07	0.00	100.00	15.25	25.59	4.22
B4-A :: PE-Cy7	0.01	0.03	0.13	0.46	2.52	100.00	0.38	8.00
R1-A :: AF647	0.04	0.05	0.00	-0.01	1.38	0.21	100.00	14.26
R2-A :: NiR	-0.40	-0.10	0.21	0.12	0.37	3.61	15.00	100.00

4.3. Data Transformation Details

4.3.1 Data Transformation Description

FlowJo Version 10 has been used for analysis and visualization of the data.

FSC and SCC were acquired and displayed with linear scaling, all fluorescence parameters were acquired with log scaling and bi-exponentially transformed for data visualization.

4.3.2 Relevant transformation details

FlowJo version 10.0.6 to 10.4 running on Windows 7 was used for transforming the data

4.4. Gating Details

4.4.1. Gate description for *ex vivo* Phenotyping

Cell type/Characteristic	Gating Hirarchy for Immune cell Populations					
Cells (PBMC)	FSC-A/SSC-A					
Single cells 1	FSC-A/SSC-A	FSC-H/FSC-W				
Single cells 2	FSC-A/SSC-A	FSC-H/FSC-W	SSC-H/SSC-W			
Live cells	FSC-A/SSC-A	FSC-H/FSC-W	SSC-H/SSC-W	FSC-A/LD NiR		
T cells	FSC-A/SSC-A	FSC-H/FSC-W	SSC-H/SSC-W	FSC-A/LD NiR	CD3+/CD19-	
B cells	FSC-A/SSC-A	FSC-H/FSC-W	SSC-H/SSC-W	FSC-A/LD NiR	CD19+/CD3-	
NK cells	FSC-A/SSC-A	FSC-H/FSC-W	SSC-H/SSC-W	FSC-A/LD NiR	CD3-/CD19-	CD56+	
NKT-like cells	FSC-A/SSC-A	FSC-H/FSC-W	SSC-H/SSC-W	FSC-A/LD NiR	CD3+/CD56+		
Treg	FSC-A/SSC-A	FSC-H/FSC-W	SSC-H/SSC-W	FSC-A/LD NiR	CD4+/CD3+	CD25hi/CD127lo	FOXP3+
CD4+ T cells	FSC-A/SSC-A	FSC-H/FSC-W	SSC-H/SSC-W	FSC-A/LD NiR	CD3+	CD4+/CD8α-	
CD8+ T cells	FSC-A/SSC-A	FSC-H/FSC-W	SSC-H/SSC-W	FSC-A/LD NiR	CD3+	CD4-/CD8α+	
CD4-CD8- T cells	FSC-A/SSC-A	FSC-H/FSC-W	SSC-H/SSC-W	FSC-A/LD NiR	CD3+	CD4-/CD8α-	
γδ T cells	FSC-A/SSC-A	FSC-H/FSC-W	SSC-H/SSC-W	FSC-A/LD NiR	CD3+/TCRγδ+		
CD161+ ILC	FSC-A/SSC-A	FSC-H/FSC-W	SSC-H/SSC-W	FSC-A/LD NiR	CD3-	SSC-A^{lo}/CD161+	
classical Monocytes	FSC-A/SSC-A	FSC-H/FSC-W	SSC-H/SSC-W	FSC-A/LD NiR	Lin (CD3/56/19/20)-/HLA-DR+	CD14+/CD16-	
intermediate Monocytes	FSC-A/SSC-A	FSC-H/FSC-W	SSC-H/SSC-W	FSC-A/LD NiR	Lin (CD3/56/19/20)-/HLA-DR+	CD14+/CD16+	
non-classical Monocytes	FSC-A/SSC-A	FSC-H/FSC-W	SSC-H/SSC-W	FSC-A/LD NiR	Lin (CD3/19/20/56)-/HLA-DR+	CD14-/CD16+	
myeloid dendritic cells (mDC)	FSC-A/SSC-A	FSC-H/FSC-W	SSC-H/SSC-W	FSC-A/LD NiR	Lin (CD3/14/19/20/56)-	CD11c+/HLA-DR+	
plasmacytoid dendritic cells (pDC)	FSC-A/SSC-A	FSC-H/FSC-W	SSC-H/SSC-W	FSC-A/LD NiR	Lin (CD3/14/19/20/56)-	CD123+/HLA-DR+	

4.4.2 Gate statistics

Count and frequency of gated population within parent population were exported and used to calculate frequency within the live cell gate. For absolute frequencies, these frequencies were applied to the live PBMC yield (live cell frequency determined by FACS was applied to the number of PBMC isolated per ml of whole blood).

4.4.3 Gating strategy for *ex vivo* phenotyping

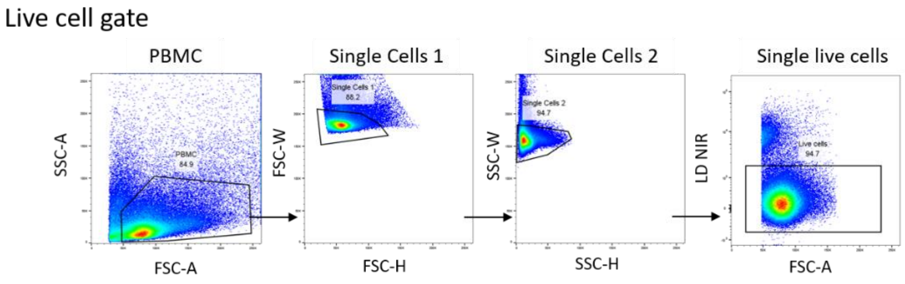

Figure 1. Live cell gating strategy

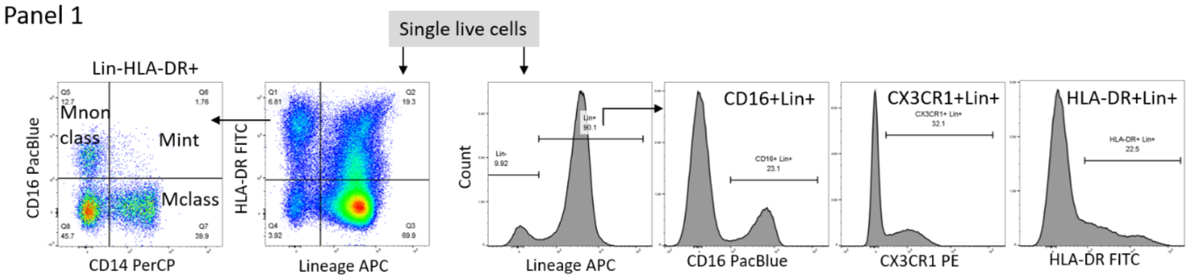

Figure 2. Panel 1: Monocyte subsets and lymphocyte activation/ cytotoxicity

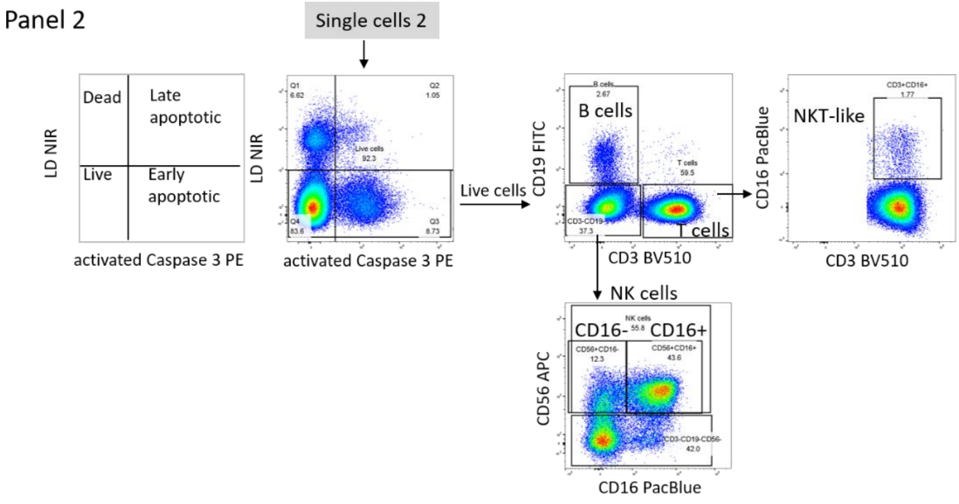

Figure 3. Panel 2: Apoptosis and lymphocyte subsets

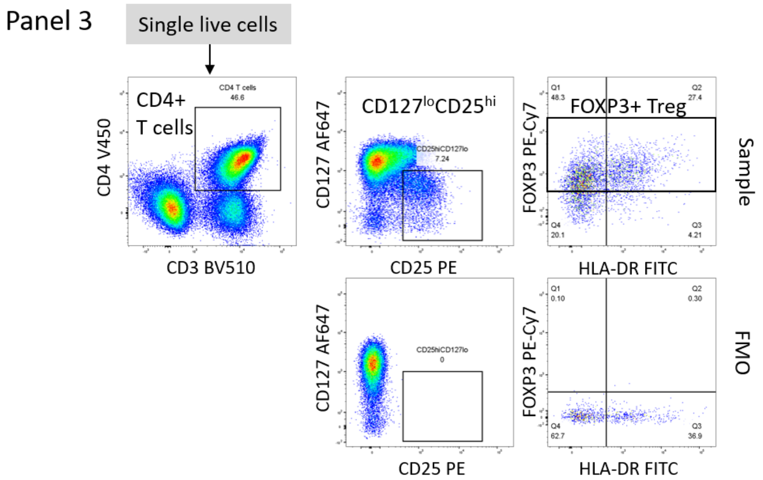

Figure 4. Panel 3: Regulatory T cells (Treg)

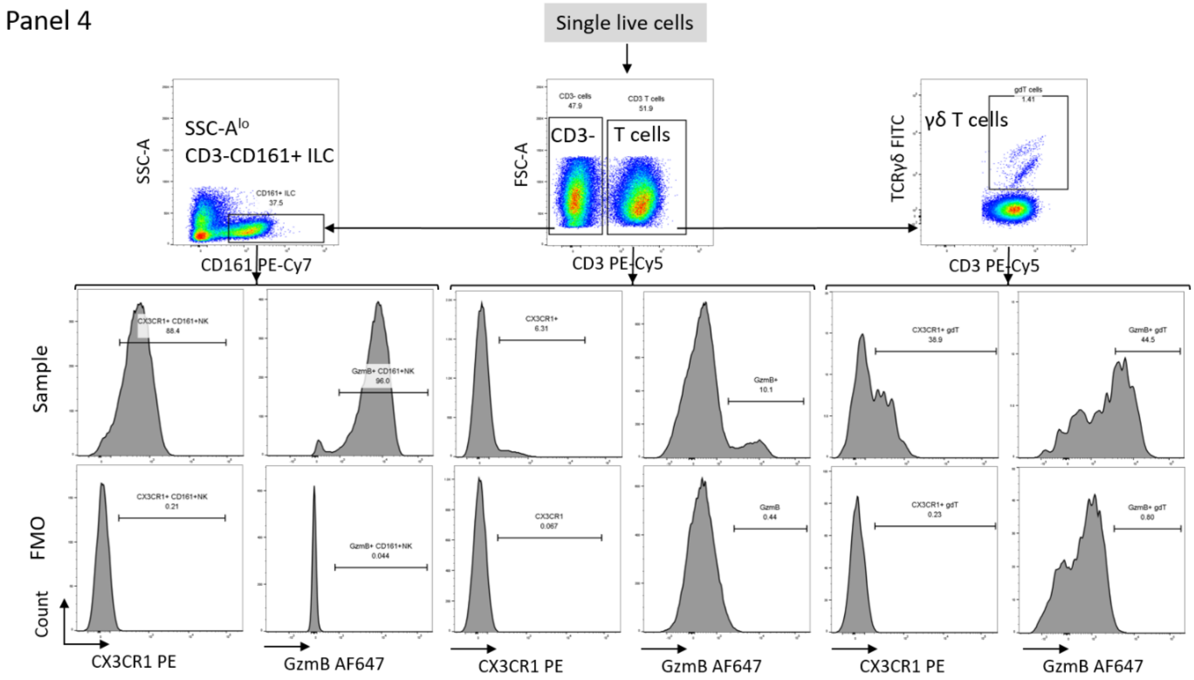

Figure 5. Panel 4: Cytotoxicity markers on T cells, γδ T cells and innate lymphoid cells (ILC)

Panel 5

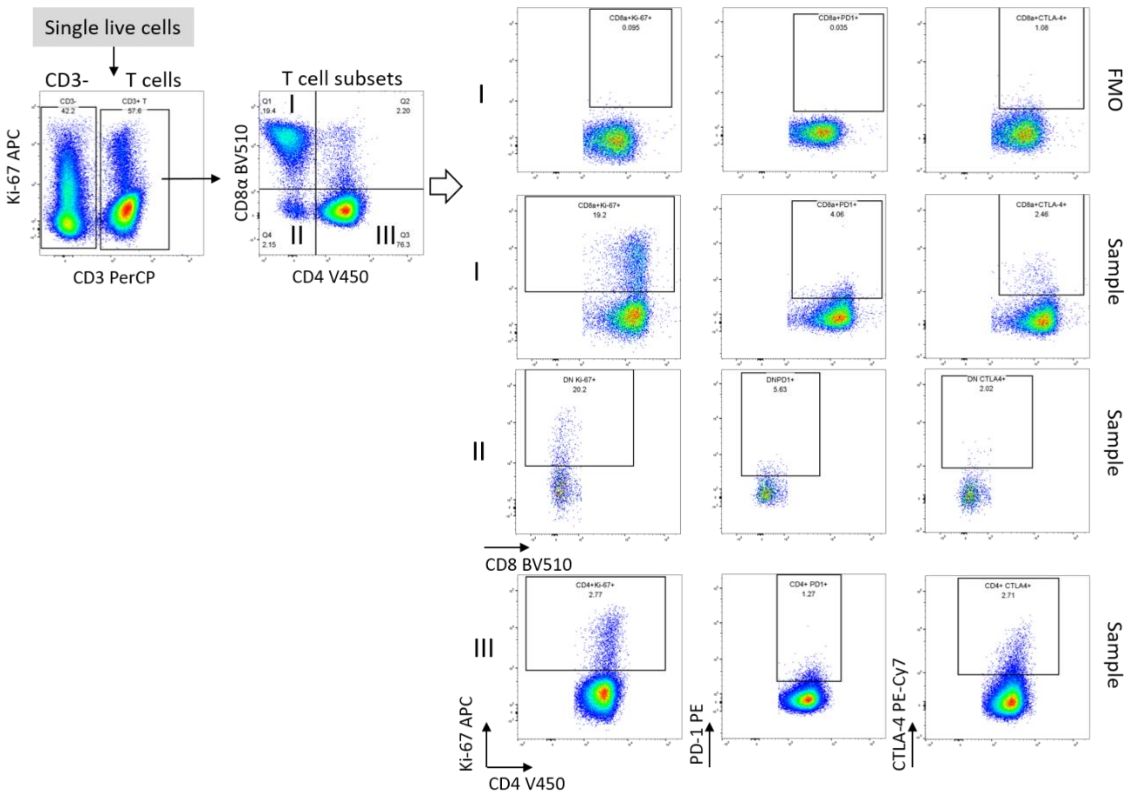

Figure 6. Panel 5: Proliferation and exhaustion in T cell subsets

Panel 6

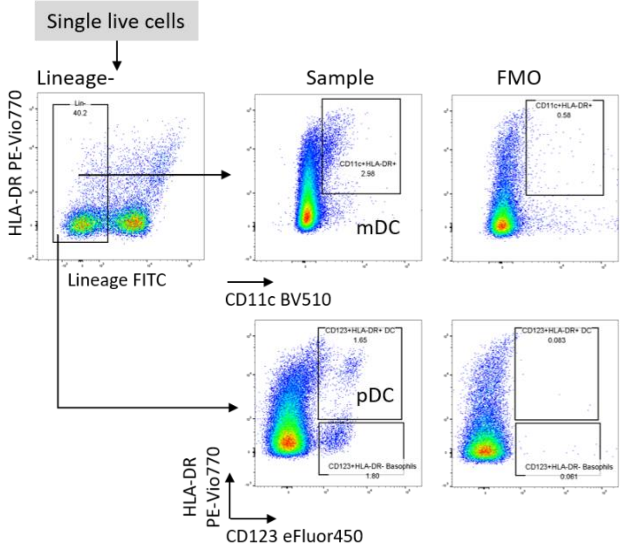

Figure 7. Panel 6: Dendritic cells

ICCK

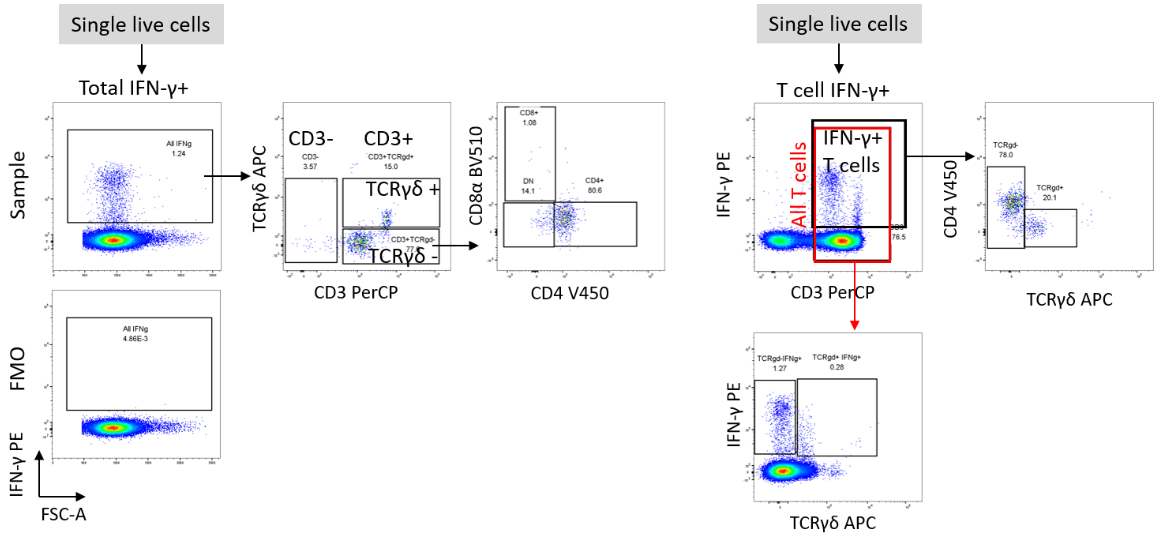

Figure 8. ICCK Panel: Composition of total IFN-γ+ cells and IFN-γ+ T cells (two different gating strategies were employed to analyse data)
